# Facile Preparation of SrZr_1-x_Ti_x_O_3_ and SrTi_1-x_Zr_x_O_3_ Fine Particles Assisted by Dehydration of Zr^4+^ and Ti^4+^ Gels under Hydrothermal Conditions

**DOI:** 10.3390/nano13152195

**Published:** 2023-07-28

**Authors:** José Remigio Quiñones-Gurrola, Juan Carlos Rendón-Angeles, Zully Matamoros-Veloza, Jorge López-Cuevas, Roberto Pérez-Garibay, Kazumichi Yanagisawa

**Affiliations:** 1 Centre for Research and Advanced Studies of the National Polytechnic Institute, Saltillo Campus, Ramos Arizpe 25900, Mexico; jose.quinones@cinvestav.edu.mx (J.R.Q.-G.); jorge.lopez@cinvestav.edu.mx (J.L.-C.); roberto.perez@cinvestav.edu.mx (R.P.-G.); 2 Tecnológico Nacional de México/(I.T. Saltillo), Technological Institute of Saltillo, Graduate Division, Saltillo 25280, Mexico; zully.mv2@saltillo.tecnm.mx; 3 Research Laboratory of Hydrothermal Chemistry, Faculty of Science, Kochi University, Kochi 780-8073, Japan; yanagi@kochi-u.ac.jp

**Keywords:** hydrothermal synthesis, gel dehydration, SrZrO_3_–SrTiO_3_, solid solution, crystallization, cubic structure, orthorhombic structure

## Abstract

In recent decades, perovskite-type compounds (ABB′O_3_) have been exhaustively studied due to their unique ferroelectric properties. In this work, a systematic study aiming to prepare fine particles in the binary system SrZrO_3_–SrTiO_3_ was conducted under hydrothermal conditions in a KOH (5 M) solution at 200 °C for 4 h under a constant stirring speed of 130 rpm. The precursors employed were SrSO_4_ powder (<38 μm size) and coprecipitated hydrous gels of Zr(OH)_4_•9.64 H_2_O (Zr-gel) and Ti(OH)_4_•4.5H_2_O (Ti-gel), which were mixed according to the stoichiometry of the SrZr_1-x_Ti_x_O_3_ in the compositional range of 0.0 > x > 100.0 mol% Ti^4+^. The XRD results revealed the formation of two crystalline phases rich in Zr^4+^, an orthorhombic structured SrZr_0.93_Ti_0.07_O_3_ and a cubic structured SrZr_0.75_Ti_0.25_O_3_ within the compositional range of 0.1–0.5 mol of Ti^4+^. A cubic perovskite structured solid solution, SrTi_1-x_Zr_x_O_3_, was preferentially formed within the compositional range of 0.5 > x > 0.1 mol% Ti^4+^. The SrZrO_3_ and SrZr_0.93_Ti_0.07_O_3_-rich phases had particle sizes averaging 3 μm with a cubic morphology. However, a remarkable reduction in the particle size occurred on solid solutions prepared with hydrous Ti-gel over contents of 15 mol% Ti^4+^ in the reaction media, resulting in the formation of nanosized particle agglomerates with a cuboidal shape self-assembled via a 3D hierarchical architecture, and the sizes of these particles varied in the range between 141.0 and 175.5 nm. The limited coarsening of the particles is discussed based on the Zr-gel and Ti-gel dehydration capability differences that occurred under hydrothermal processing.

## 1. Introduction

Perovskite structured oxide compounds with an ABX_3_ chemical formula have been investigated because of their broad practical applications and academic research interest. These are associated with their unique properties due to their relatively simple crystalline structure [1,2,3]. The cubic structural perovskite framework (*Pm3m*) comprises two linked coordination polyhedral networks, the BX_6_ octahedra and AX_12_ cuboctahedra. Bonding between the BX_6_ octahedra polyhedron and sites incorporating A cations occurs via corner sharing. Interestingly, the BX_6_ octahedra exhibit volumetric variations, namely expansion, contraction, and tilt, to maintain non-ideal ionic size substitutions. Likewise, the local electronic instabilities can distort the octahedra or cause a slight cation shift from their unit cell structural positions. Perovskite structured materials allow ionic partial substitutions and the formation of vacancies on their three structural atomic locations [3]. Therefore, this structure is remarkably flexible to locate various periodic table elements at both A and B sites, resulting in a wide range of properties. The most important are ferroelectricity, ferromagnetism and colossal magnetoresistance, piezoelectricity and multiferroicity [3,4]. B-site cation doping has been one of the major research subjects in developing new advanced perovskite materials; this site controls and tunes some of the most technologically attractive properties, such as electrical conductivity and magnetic order [5,6,7,8]. The tuning of these properties results from combining at least two elements, which maintain the cation structural ordering allowing to development of advanced new materials [3].

The perovskite binary system SrTiO_3_–SrZrO_3_, which has technological importance due to its dielectric properties [9], has attracted significant research interest for solid solution preparation via the B-site cation isomorphous substitution. The end members of this system, cubic SrTiO_3_ (space group *Pm3m*) and orthorhombic SrZrO_3_ (*Pbnm*) do not possess ferroelectric properties. However, the partial substitution of Zr^4+^ up to 5 mol% provided a remarkable increase in the dielectric breakdown strength (14.4 kV/mm), simultaneously maintaining the high dielectric constant (330) characteristic of SrTiO_3_, which makes these materials suitable for technical applications as high voltage capacitors [9,10]. Likewise, the SrTi_1-x_Zr_x_O_3_ microstructure mobility causes octahedral structural transitions, which consequently cause electronic changes, resulting in a peculiar ferroelastic switching behavior; this feature is essential for magnetic applications [11,12,13]. Similarly, the superlattice crystalline structures formed in SrTi_1-x_Zr_x_O_3_ layered compounds achieve special BX_6_ ordering that induces a two-dimensional tension mechanism, causing, at low temperatures, a ferroelectric Ti-rich solid solution (SS) Ti [13]. To some extent, the structural distortion somewhat might promote the proton motion [14]. Another potential technical application determined for ultrafine powders of SrTi_1-x_Zr_x_O_3_ solid solutions SS include photoluminescence emission [15] and organic dye photocatalysts [16].

Various systematic studies have been focused on the SS-SrTi_1-x_Zr_x_O_3_ preparation in the 0.0 ≥ x ≤ 0.5 mol% Zr^4+^ compositional range by the conventional high-temperature solid-state reaction [1,4,6,8,10,11]. The powders were produced by mixing high-purity SrCO_3_, TiO_2_ and ZrO_2_ powders, which were appropriately mixed in stoichiometric molar ratios to form a slurry with acetone. The powder mixtures were homogenized by vigorous grinding and then pressed. The pellets underwent calcination in a three-step heat treatment between 800 and 1400 °C for 24 to 96 h reaction intervals. However, the SS-SrTi_1-x_Zr_x_O_3_ monodisperse nanoparticle formation was not feasible under the conventional ceramic processing route.

On the contrary, chemical solution processing techniques, namely sol-spray pyrolysis [5], low-temperature coprecipitation [7], and sol–gel [16], successfully achieved the preparation of SrTi_1-x_Zr_x_O_3_ nanoparticles with accurate stoichiometric compositions, controlled morphology and monomodal particle size distributions that boosted their physical and chemical properties [5,7,16]. Furthermore, the hydrothermal technique has successfully prepared SrTiO_3_ mesocrystals with nanoparticle sizes and controlled cubic morphology [17,18,19]. The systematic studies focused on the chemical reaction of a slurry media containing TiO_2_ particles and Sr(OH)_2_ hydrolyzed in a highly alkaline NaOH media (5–10 M) under hydrothermal conditions, resulting in the formation of the SrTiO_3_ nanoparticles via two mechanisms, namely the topochemical transformation of the TiO_2_ particles [17,18] and the conventional dissolution–precipitation process [19]. A different approach involving the employment of a mineral source as Sr^2+^ (SrSO_4_) precursor and hydrous Ti-gel (Ti(OH)_4_•4.5H_2_O) was also evaluated under hydrothermal conditions at 250 °C for 24 h in a KOH solution 5 M [20]. Under these conditions, the dissolution of the SrSO_4,_ coupled with the Ti-gel dehydration, established the chemical equilibrium for triggering the crystallization of monodispersed fine cubic-shaped SrTiO_3_ crystals, which exhibited a monomodal particle size distribution. This process can reduce global production costs compared to the low-temperature chemical solution processing and the conventional hydrothermal technique, which uses pure Sr^2+^ chemical reagents produced from the celestite mineral.

A literature review suggests that further efforts must be made to investigate the feasibility of producing SrZr_1-x_Ti_x_O_3_ or SrTi_1-x_Zr_x_O_3_ solid solution via hydrothermal alkaline processing. Hence, this work systematically investigated the preparation of particles with different chemical compositions in the binary SrZrO_3_-SrTiO_3_ system in alkaline media (5 M KOH). The experiments were conducted at a standard temperature (200 °C), which is relatively low for rapidly dissolving the SrSO_4_ particles, and seemingly vertical agitation might provide an adequate dispersion of the mineral particles and precursor gels in the alkaline solvent solution during the treatment. Particular emphasis was on determining the effect of the precursor gels (Zr-gel and Ti-gel) dehydration capability on the particle crystallization of both SrZr_1-x_Ti_x_O_3_ and SrTi_1-x_Zr_x_O_3_ (SS). A detailed analysis was made to determine the stability of the tetragonal structure in the compositional 2.5 ≥ x ≤ 50.0 mol% of Ti^4+^ for the SrZr_1-x_Ti_x_O_3_ samples. The nanoparticle size and morphological differences were correlated with the dehydration capability of the gels.

## 2. Materials and Methods

### 2.1. Materials and Preparation of Zr^4+^ and Ti^4+^ Precursor Gels

Reagent-grade zirconium IV (ZrCl_4_) and titanium IV (TiCl_4_) chlorides (Wako, Japan, 99.0% purity) were used for preparing the precursor gels. Initially, the transition metal precursor solutions were produced with a concentration of 0.43 M for ZrCl_4_ and TiCl_4_ with deionized water_,_ following the procedure described elsewhere [20,21], respectively. A 500 mL liquor aliquot of each solution was poured into a glass beaker, and then the gels were precipitated by adding a volume of 150 mL of 5 M NaOH solution. Each gel was separated from the residual mother liquor by centrifugation. The thermogravimetry analysis determined the total weight loss of each gel, indicating that the chemical composition of the hydrous gels corresponds to Zr(OH)_4_•9.64H_2_O and Ti(OH)_4_•4.5H_2_O, respectively. The SrSO_4_ mineral (Celestite) crystals were previously grounded and pulverized to a particle size < 38 μm. The wet chemical analysis of the highly pure crystalline SrSO_4_ (1 g) revealed that the mineral is constituted by 46.61 wt% Sr, 1.33 wt% Ba, and 52.05 wt% SO_4_^2-^, which correspond to 96.8 wt% SrSO_4_ and 2.25 wt% BaSO_4_, plus the following minor compounds were CaO (0.73 wt%), Fe_2_O_3_ (0.19 wt%), MnO (0.02 wt%) and Al_2_O_3_ (0.007 wt%). The alkaline reagent-grade chemical employed was KOH (Wako, Japan, 99.0% purity).

### 2.2. Hydrothermal Treatments

**Figure 1:**
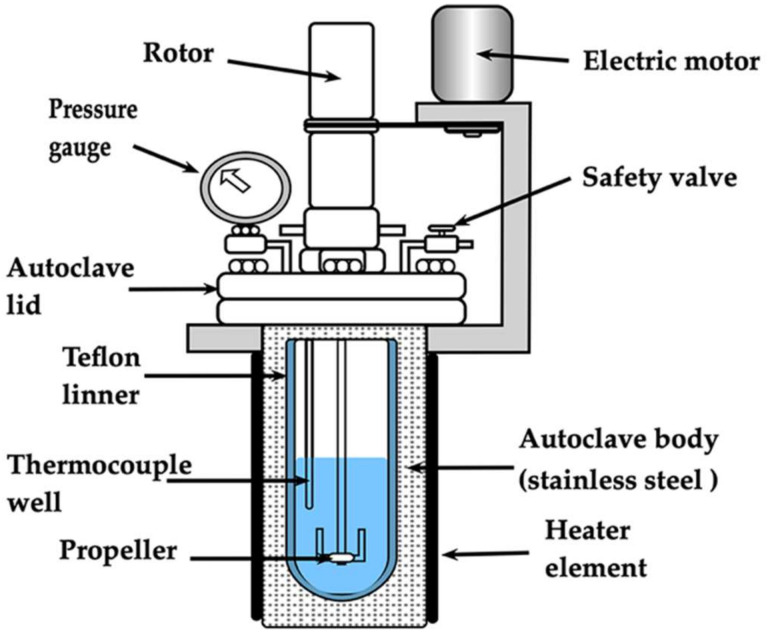
Scheme of the stainless-steel vessel accoupled with a vertical stirring propel and internal Teflon liner tube with a capacity of 500 mL.

Experiments aimed to determine the feasibility of producing stable SrZr_1-x_Ti_x_O_3_ and SrTi_1-x_Zr_x_O_3_ and to elucidate the solid solution (SS) crystalline structural and microstructural features, which might vary due to transition metal gel dehydration process that occurs under alkaline stirring hydrothermal conditions. Other experimental parameters study, such as the reaction time and temperature, were avoided in the present study. Therefore, the synthesis of the solid solutions in the binary SrZrO_3_–SrTiO_3_ system proceeded in the entire compositional range. However, a particular emphasis took place on the molar content of 1.0 ≥ x ≤ 0.5 of Zr^4+^ to investigate the tetragonal structure stability; this crystalline structure is predominant at temperatures lower than 400 °C [5,10]; thus, the compositions studied varied in a molar span of 2.5 mol% Zr^4+^. A SrSO_4_ powder sample (4.8 ± 0.05 g) was placed at the bottom of a Teflon test tube-type chamber (500 mL volume), the amount of each gel was calculated according to the chemical formulas mentioned in Section 2.1, and the stoichiometric Zr:Ti molar ratio 1-x:x, was then added. All the treatments were conducted at a constant volume (95 mL) of the 5 M KOH solution selected as a solvent media. The stainless-steel autoclave was sealed, and the vertical propel rotation set was at 130 rpm during the heating and soaking stages (Figure 1). The autoclave was then heated at a constant speed of 5 °C/min up to the standard temperature of 200 °C, which has been proposed as optimum in preliminary research kinetics studies focused on synthesizing SrTiO_3_ [20] and SrZrO_3_ [21] under similar hydrothermal conditions. The treatments were conducted at this temperature for 4 h. After treatment, the reaction products were separated from the remaining mother liquor and ultrasonically washed several times with hot water. Then, the powders were dried at 80 °C overnight.

### 2.3. Characterization

Powder X-ray diffraction (PXRD). The analyses were conducted in a Rigaku Ultima IV diffractometer operated at 40 kV and 20 mA, using Cu-Kα radiation (*λ* = 1.54056 Å). XRD analyses were collected in the 2θ range of 5–80° at a constant scanning speed of 20°/min with a 0.02° step. Rietveld refinement analyses were carried out on the PXRD patterns collected in the 2θ range of 15–130°, under standard conditions at a scanning speed of 0.01°/min and 0.002° step. The refinement calculations were performed using the TOPAS 4.2 software (Bruker AXS: Karlsruhe, Germany, 2009). The space group, and the atomic position (Wyckoff number and coordinates), correspond to the COD card No. 96-900-7150. All details associated with the Rietveld refinement analysis are in the Supplementary Supporting Information File (hereafter referred to as SSIF), Section S1 includes Tables S1 and S2 where the Wyckoff spatial coordinates are given.

Morphology and microstructural observation. The microstructural aspects of the produced particles were observed in a field emission scanning electron microscopy (JEOL JSM-7100F) equipped with a solid-state microprobe (EBSD, Bruker e-flash). Typical micrographs were observed with the microscope operated at 10 kV and a constant current of 69 μA. The particle size distribution was statistically determined from SEM images of 50 particles. Crystalline structural details of selected SrZr_1-x_Ti_x_O_3_ particles were revealed using high-resolution transmission electron (HR-TEM, Philips Titan 300) operated at 200 kV.

Differential scanning calorimetry analysis (DSC). The DSC technique was applied to determine the thermal behavior of single Zr-gel and Ti-gel and some selected samples with varied Zr/Ti weight ratios. These analyses also revealed the differences in the dehydration behavior of the Zr/Ti raw gel mixtures. Simultaneously, the gel weight loss of selected samples was determined by thermal gravimetric analyses (TGA). These analyses were carried out using a Perkin Elmer Pyris Diamond TG/DSC apparatus in the temperature range from 30 to 1000 °C. The heating rate selected for the thermal evaluation was 10 °C/min; all the treatments were conducted using an air atmosphere.

## 3. Results

### 3.1. Thermal Stability of Gel Mixtures and Zr-Gel and Ti-Gel

**Figure 2:**
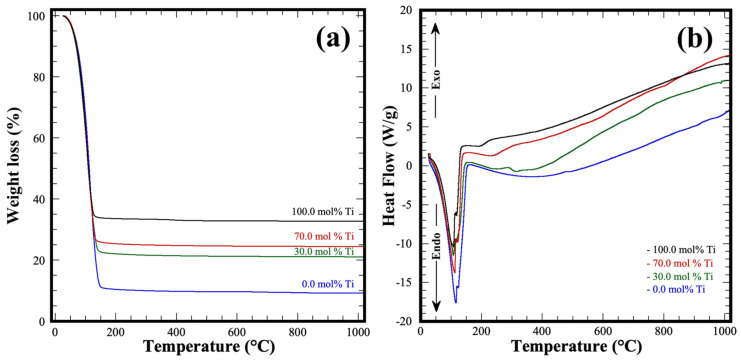
(**a**) Gels weight loss variation determined via TGA analyses, and (**b**) thermal behavior of the precursor Zr-Ti gels determined by DSC in air atmosphere up to 1000 °C.

Thermogravimetry and DSC analyses were conducted to evaluate the thermal stability and structural changes of single pasty Zr-gel, Ti-gel and some selected gels mixtures containing different Zr/Ti ratios. These analyses aimed to determine the total weight loss monitored in the air atmosphere within the 30–1000 °C temperature range. The change in the weight of the gels is portrayed in Figure 2a. In general, the gels selected exhibited a remarkable weight loss above 50 °C, without further weight variation above 150 °C. Interestingly, the Zr pure gel had the most significant weight loss of approximately 90.93 wt%; in comparison with that observed for the Ti pure (67.2 wt%) and Zr/Ti (75.5–80.0 wt%) gel samples. According to the total amount of water that Zr and Ti pure gels released during the heat treatment, their chemical formulas are Zr(OH)_4_•9.64H_2_O and Ti(OH)_4_•4.5H_2_O, respectively.

Furthermore, the thermal behavior of all the Zr-Ti gels observed in the DSC curves (Figure 2b) revealed an endothermic event in the temperature range from 45–150 °C. This event is irrespective of the pasty gel chemical composition; therefore, this peak correlates with the water molecules’ ultimate dehydration process. Above 200 °C, only a regular baseline variation was observed on all samples rather than the expected crystallization events associated with the oxide formation on all the gels. Moreover, the differences in the dehydration degree determined might alter the bulk concentration of the alkaline fluid used during the hydrothermal treatments conducted under the standard hydrothermal conditions of 200 °C for 4 h under stirring. This agrees with the differences in the crystal growth of the SrZrO_3_ particles hydrothermally synthesized using a pasty Zr-gel and a previously dried amorphous Zr(OH)_4_^0^ reported elsewhere [21].

### 3.2. Structural Aspects of the Hydrothermal Synthesis of SrZr_1-x_Ti_x_O_3_ and SrTi_1-x_Zr_x_O_3_ SS

**Figure 3:**
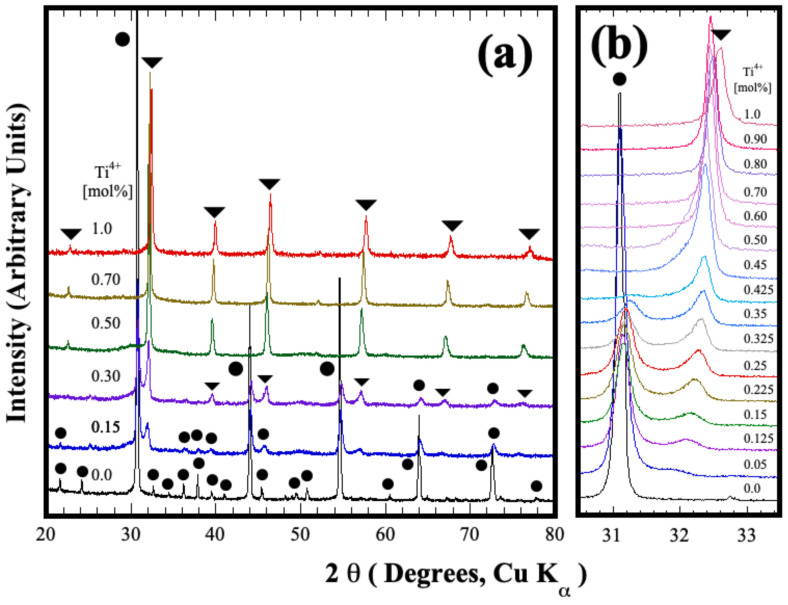
(**a**,**b**) X-ray diffraction patterns of the products obtained hydrothermally by treating celestite powders with various molar contents of pasty Zr(OH)_4_•9.64H_2_O and Ti(OH)_4_•4.5H_2_O gels, with a solvent (5 M KOH) volume filling ratio of 20% at 200 °C for 4 h. Perovskite single phases: (●) orthorhombic SrZrO_3_, and (▼) cubic SrTiO_3_.

Figure 3 shows the typical XRD patterns of selected powders produced under hydrothermal conditions at 200 °C for 4 h under constant stirring (130 rpm), varying the content of Ti^4+^ in the mixture. Generally, the single-step chemical reaction between the SrSO_4_ and the pasty gels is enhanced under stirring conditions, triggering the formation of a fine white powder, as confirmed in all treatments by naked-eye observations. When the Zr-gel was solely used as a precursor of the tetravalent metal perovskite, the peaks in the XRD pattern were indexed by those of the SrZrO_3_ perovskite with orthorhombic structure (ICDD 70-0283 space group *Pbnm,* Figure 3a). On the contrary, at Ti^4+^ contents over 10.0 mol%, new peaks located at 2θ angles of 32.25°, 39.7°, 45.8° and 56.9° were determined in the PXRD pattern, as seen in Figure 3a. To determine the formation pathway of the new compound, experiments were conducted with a Ti^4+^ compositional span variation of 2.5 mol% Ti^4+^. The new phase was formed under alkaline hydrothermal conditions in the Ti^4+^ compositional range between 7.5 and 47.5 mol% Ti^4+^, as depicted by the signal that appeared at 32.25° 2θ angle (see Figure 3b). It deserves emphasizing that this peak exhibited a gradual shift toward higher 2θ angles coupled with peak intensity increment. These crystalline structural features are likely due to the Ti^4+^ gradual incorporation into the crystalline structure, and its bulk content produced over 7.5 mol% Ti^4+^ contents in the reaction media. Simultaneously, the central peak corresponding to the orthorhombic structured SrZrO_3_:Ti^4+^ (hereafter referred to as SS1) did not exhibit further compositional variations because it remained at a mean 2θ angle of 31.09°. Therefore, these results suggest that the chemical composition of this solid solution is SrZr_0.925_Ti_0.075_O_3_. However, the content of this stable phase was proportionally reduced by increasing the Ti^4+^ amount, as indicated by the progressive reduction in the orthorhombic phase peaks in the PXRD pattern (Figure 3a). Above 50.0 mol% Ti^4+^ peaks corresponding to a new secondary phase (hereafter referred to as SS2) were obtained. Based on these structural results, we infer that the crystalline structure of the SS2 single-phase can be indexed with the cubic structured SrTiO_3_ perovskite (space group *Pm3m*, ICDD card no. 40-1500) because it agrees with the peaks distribution of the SrTiO_3_ single pattern. These results depict that the cubic perovskite SS2 crystalline phase is chemically stable under hydrothermal conditions in the compositional range of 7.5–100.0 mol% Ti^4+^. In addition, the powder crystallized without contaminant by-products, namely SrCO_3_, which is predominantly formed due to its low solubility in alkaline solutions [22]. Furthermore, a steady-state chemical reaction involving the solute saturation of the hydrothermal media is likely to proceed and trigger the simultaneous crystallization of both crystalline compounds under fluid stirring [23].

**Figure 4:**
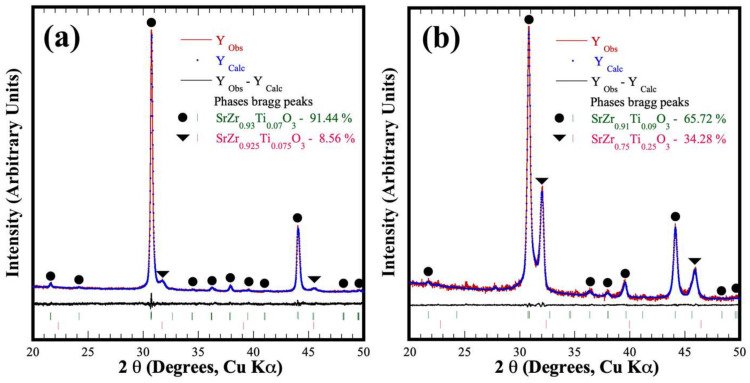

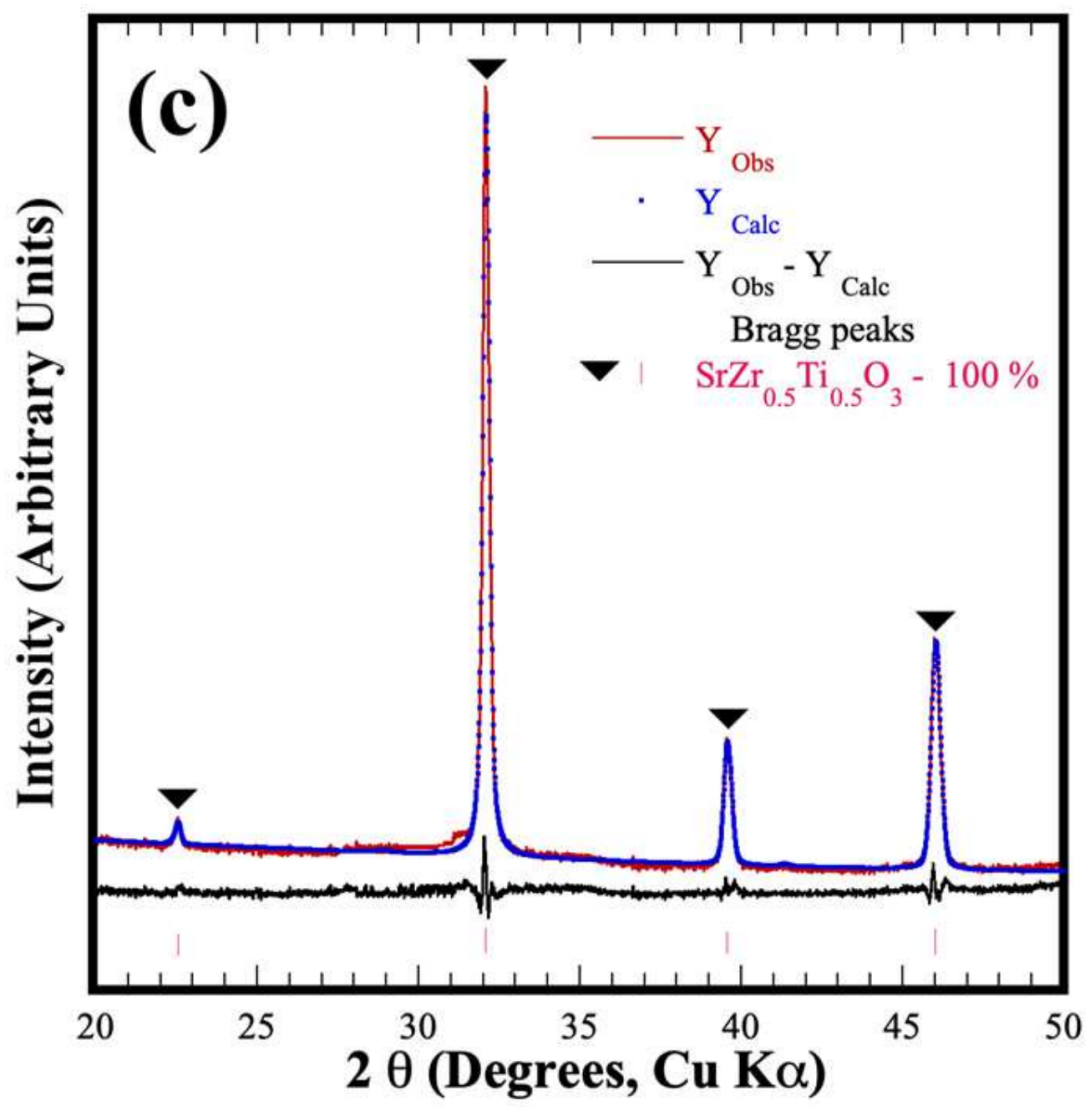
Rietveld refinement plots of products obtained hydrothermally using celestite powders mixed with different metal precursor gels in molar ratios of (**a**) 92.5:7.5, (**b**) 75.0:25.0 and (**c**) 62.5:37.5 mol% Zr^4+^:Ti^4+^, with a solvent (5 M KOH) volume filling ratio of 20% at 200 °C for 4 h. Perovskite single phases (●) orthorhombic and (▼) cubic.

The compositional variation suggested by the PXRD analyses was observed in the SS2 phase. Stoichiometric computation was carried out to determine the Ti^4+^ content incorporated in the cubic perovskite secondary SS2’ phase (new phase produced in the Ti^4+^ range of 7.5–47.5 mol%) and into the orthorhombic SS1; this procedure considered the nominal Ti^4+^ molar content added as a raw material. Likewise, the fraction of each solid solution that constitutes the ultimate powder produced was calculated by the expression [(1-x)SS1 + xSS2′] = 1, which depicts the quantitative variation in the phase content revealed by the diffraction patterns of Figure 3b. The typical Rietveld refined plots that correlate the quantitative stoichiometric computation abovementioned are shown in Figure 4. Interestingly, a minimal variation in the residual difference was also revealed in those samples exhibiting the formation of SS1 and SS2 perovskite phases. These results show that the calculated Zr^4+^:Ti^4+^ stoichiometric contents in the orthorhombic and cubic phases with *Pbnm* and *Pm3m* space groups highly fitted the atomic occupation in the Rietveld refinement algorithm. The algorithm ultimately computed the fraction content of each perovskite phase in the powder sample prepared with nominal molar Zr^4+^:Ti^4+^ ratios of (a) 92.5:7.5 (b) 75.0:25.0 and (c) 62.5:37.5, as it can be seen in Figure 4. Additional results are shown in the refinement plots in Figure S1 and the unit cell lattices in Figure S2 in the SSIF. Furthermore, the structural crystalline refinement approach also considered other fitting parameters such as background, thermal isotropy, lattice parameters, scale factor, profile half-width, crystallite size and local strain; the details regarding the atomic Wyckoff elemental spatial distribution are given in the SSIF, which includes Table S3 (see the SSIF) that summarizes the results of the crystalline structural refinement. This approach was adequate to fit the structural differences of the reaction products obtained after the hydrothermal reaction, which is depicted by both the low mean values of the goodness-of-fit factor (GOF, mean χ^2^ value of 3.48%) and *R_wp_* = 3.26. It deserves to emphasize that the analyses confirmed that the orthorhombic and cubic crystalline perovskite particles were simultaneously formed under hydrothermal conditions employing the highly soluble SrSO_4_ powder and the mixture of Zr^4+^ and Ti^4+^ gels.

**Figure 5:**
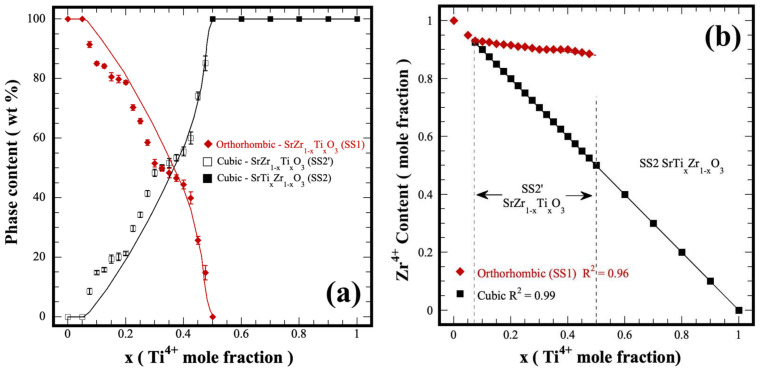
(**a**) Content variation in the hydrothermally prepared solid solutions SS1, SS2′ and SS2, and (**b**) change in the Zr^4+^ molar fraction in both the orthorhombic (SS1) and cubic (SS2′) in the compositional range of 7.5–47.5 mol% Ti^4+^ of the binary system SrZrO_3_-SrTiO_3_.

The variation in the content of both orthorhombic and cubic structured solid solutions, which were hydrothermally produced within the whole compositional range of the binary system SrZrO_3_-SrTiO_3_; using a KOH 5 M solution, is portrayed in Figure 5a. Generally, the formation of the new cubic perovskite phase (SS2′) occurred in the compositional range between 7.5 mol% and 47.5 mol% Ti^4+^. The quantitative analyses conducted on the nominal stoichiometric tetravalent metal compositions depict that the new cubic SS2′ phase reach in Zr^4+^ (SrZr_1-x_Ti_x_O_3_) exhibited a marked increase in its content when the Ti^4+^ concentration increased progressively in the solvent fluid. Interestingly, the variation in the bulk content of this phase likely resembles a typical sigmoidal kinetic behavior. On the contrary, the residual content of the hydrothermally crystallized orthorhombic phase (SS1) powder proportionally decreased following a reverse sigmoidal behavior. The simultaneous crystallization of both perovskite phases is likely triggered under hydrothermal conditions because the continuous gel dehydration promotes a cooperative reaction steady state; thereby, a constant increase in the Ti^4+^ mass gradient provoked a rise in the cubic SS2′ single phase amount. This inference is supported by the fact that the Zr^4+^ atomic ratio in the SS2’ cubic phase exhibited a linear decrease, proportional to the rise of the Ti^4+^ content, as seen in Figure 5b.

Furthermore, the orthorhombic SS1 phase had 4.0 mol % in Zr^4+^ reduction within the compositional range where both crystalline phases coexist in the binary system SrZrO_3_-SrTiO_3_. According to these compositional results, three chemical reaction equilibria drive the crystallization of chemically stable perovskite SS in the hydrothermal system studied. These are included in the Supplementary Supporting Information File. The chemical formulas of all the SS1, SS2′ and SS2 hydrothermally prepared are summarized in Table S3 in the SSI file, and the contents of the phases SS1 and SS2′ are also included.

**Figure 6:**
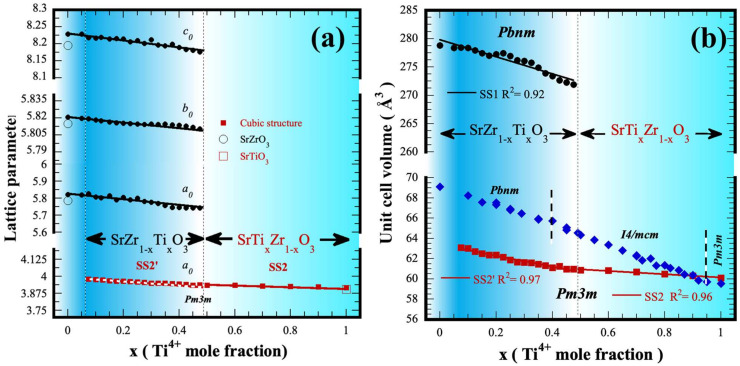
Variation in the crystalline structural parameters for the orthorhombic (SS1) and cubic (SS2′ and SS2) perovskite structured solid solutions prepared under hydrothermal conditions at 200 °C for 4 h with stirring at 130 rpm. (**a**) Unit cell lattice parameters and (**b**) unit cell volume. ♦ Unit cell values of SrZr_1-x_Ti_x_O_3_ and SrTi_1-x_Zr_x_O_3_ solid solutions produced at a high temperature within the entire compositional range of the binary system SrZrO_3_–SrTiO_3_; data taken from [8].

Additionally, details on the crystalline unit cell, lattice parameters and volume cell, corresponding to the perovskite solid solutions, which were produced under hydrothermal conditions in a 5 M KOH solution at 200 °C for 4 h with a constant stirring speed of 130 rpm, are portrayed in the graphs in Figure 6. Generally, the lattice constants corresponding to the end members of the perovskite binary system SrZrO_3_-SrTiO_3_ are slightly higher in comparison with the values reported in Table S3 (see SSIF) for the same compounds. Meanwhile, a marked continuous decrease in “*a*_0_”, “*b*_0_”, and “*c*_0_” parameters was determined in the orthorhombic solid solution (SS1, ●), with *Pbnm* space group, by increasing the Ti^4+^ content uptake in the resulting crystalline particles, as seen in Figure 6a. Likewise, the “*a*_0_” lattice parameter corresponding to the new cubic SS2′ and SS2 (■) constituents (space group *Pm3m*) exhibited a linear decrease within the Ti^4+^ compositional variation, which occurred within the ranges of 7.5 ≥ x ≤ 50.0 mol% Ti^4+^ and 50.0 ≥ x ≤ 100.0 mol% Ti^4+^, as seen in Figure 6a. In both cases, the variation determined for lattice parameter values of either orthorhombic or cubic perovskite structures agree with the systematic peak displacement determined in the X-ray results shown in Figure 3. Interestingly, all the lattice constants calculated exhibited a linear variation, which is depicted by the mean data dispersion coefficient R^2^ that varied between 0.912 and 0.987. This behavior indicates a linear dependence of the structural parameters occurred by incorporating Ti^4+^ content in the orthorhombic and cubic structured perovskite compound produced under hydrothermal conditions. Furthermore, the linear variation in the lattice parameters agrees with Vegard’s law, despite two simultaneous perovskite-related compounds crystallizing within the compositional range of 7.5 ≥ x ≤ 50.0 Ti^4+^ mol%. Therefore, in the context of the chemical composition, the processing approach investigated is devoted to determining the chemical reaction pathway related to the formation of stable perovskite solid solution compounds in the binary system SrZrO_3_-SrTiO_3_; the results suggest that a steady-state single-step reaction is triggered under stirring hydrothermal condition, preferentially crystallize two solid solutions at the B site of the perovskite SrZr_1-x_TiO_3_ with orthorhombic and cubic structures. The reaction pathway is likely controlled by the tetravalent metal gel dehydration process inherent to the proposed system.

In addition, a similar trend was observed for the lattice constants variation for the unit cell volume of the perovskite solid solutions. These values are portrayed in the plot shown in Figure 6b, and this plot also includes the unit cell volume values calculated for the SrTi_x_Zr_1-x_O_3_ (solid ♦ symbol) solid solutions prepared at 1400 °C for 96 h [8]; these data were included for comparison purposes. Some data are likely similar to those determined in our case, namely at Ti^4+^ contents above ≤90.0 mol%. The authors demonstrate that the formation of stable solid solution preferentially occurs within the entire compositional range of the system SrZrO_3_-SrTiO_3_, as depicted by the data plotted in Figure 6b. Exhaustive crystalline structural analyses conducted by neutron and synchrotron diffraction confirmed the dependence of the SS´s composition with the crystalline structural transformations, which leads to obtaining orthorhombic, tetragonal and cubic structures stable at specific Ti^4+^ contents incorporated into the SS SrTi_x_Zr_1-x_O_3_. Superlattice reflections revealed near 2θ = 33 and 41° (021 and 122/212 Miller indexes) corresponding to the orthorhombic structure were detected up to 40.0 mol% Ti^4+^; above this content, tetragonal structure predominates as sole phase produced at high temperature, the new reflection at 2θ ≈ 40° (121) confirmed the formation of SrTi_x_Zr_1-x_O_3_ SS that belongs to this structure, which predominates at the highest Ti^4+^ content of 90.0 mol% at low temperature [8].

Interestingly, the PXRD analyses conducted on the hydrothermally prepared specimens with Ti^4+^ contents between 7.5 mol% and 90.0 mol% did not reveal the superlattice peaks associated with the perovskite tetragonal structure, despite the observation of a similar variation trend on the unit cell volume of both reaction products with orthorhombic and cubic structures prepared here, compared with the continuous solid solutions obtained via solid-state reaction at high temperature [8]. We infer from these results that the Ti-gel dehydration yield plays an essential role in achieving a steady-state chemical reaction, which enhances a specific solute supersaturation stage that causes the simultaneous crystallization of both phases. This process proceeds faster than the solid-state reaction producing a broad compositional series of solid solutions in the system SrZrO_3_–SrTiO_3_ [8].

On the other hand, according to the crystalline structural results calculated by Rietveld refinement given in Table S3 of the SSIF, the minor structural differences revealed on the unit cell parameters in both perovskite compounds (orthorhombic and cubic) are caused by the variation in the Sr-O, Ti-O and Zr-O bond lengths. A systematic increase in the Ti-O bond length proportionally occurred by decreasing the Ti^4+^ content incorporated into the octahedral BO_6_ site in the cubic structured SS2, in the compositional range of 7.5 ≤ x ≤ 90 mol% Ti^4+^. Similarly, the Sr-O bond length is considerably affected, reaching a maximum value of 2.822 Å, which is remarkably more extensive than that for the cubic structured SrTiO_3_ single phase (2.769 (1) Å, as seen in Table S3). From these results, we surmise that this structural phenomenon maintains the cubic structured solid solution thermodynamically stable at low contents of Ti^4+^ below 50.0 mol%. This inference is supported by the fact that under solid-state reaction conditions, a series of intermediate tetragonal structured solid solutions were successfully formed in a shorter range of Ti^4+^ (40.0 ≤ x ≤ 90.0 mol% Ti^4+^). It deserves to emphasize that the tetragonal structure remains stable because it corresponds to the primitive cell of the orthorhombic structured perovskite. Therefore, this structural transformation is favored by a slight structural tilt in the BO_6_ octahedra caused by the Ti^4+^ partial incorporation. This structural transformation is markedly provoked under solid-state reaction conditions at high temperatures [8]. Another factor that might cause the predominant formation of the SS2 solutions with cubic structure and their structural variations is associated with the processing conditions (reaction temperature and stirring speed), as suggested elsewhere [21]. This inference is likely supported by the marked differences determined for the lattice strain caused in the unit cell by partially incorporating Ti^4+^ in the cubic SS2′ SS. Large lattice strain values were calculated from the Rietveld refinements for the SS2′ SS prepared with Ti^4+^ contents between 7.5 and 50 mol% and are shown in Table S3 of the Supplementary Supporting Information File.

### 3.3. Particle Morphology Variation for the Hydrothermally Synthesized SrZr_1-x_Ti_x_O_3_ and SrTi_1-x_Zr_x_O_3_ (SS)

**Figure 7:**
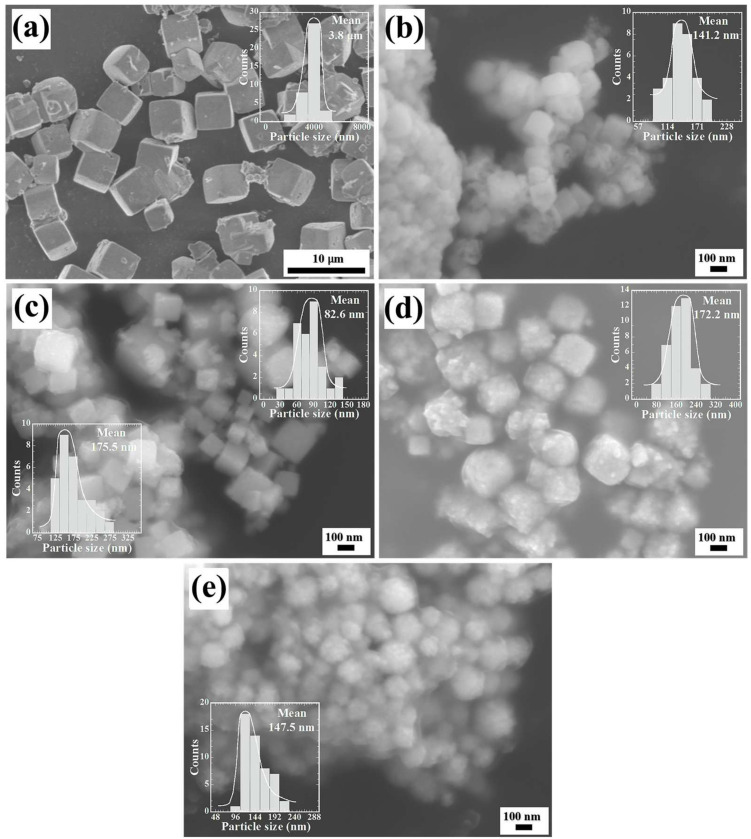
FE-SEM micrographs of the particles of the SrZr_1-x_Ti_x_O_3_ and SrTi_1-x_Zr_x_O_3_ solid solutions, obtained under dynamic hydrothermal conditions at 200 °C in a reaction medium of 5 M KOH for a reaction time of 4 h, using SrSO_4_ and different ratios of Zr and Ti gels (Zr(OH)_4_•9.64H_2_O, Ti(OH)_4_•4.5H_2_O). (**a**) SrZrO_3_, (**b**) 5.0 mol% Ti^4+^, (**c**) 30.0 mol% Ti^4+^, (**d**) 50.0 mol% Ti and (**e**) SrTiO_3_.

Recently, the morphological habit features for SrZrO_3_ particles were elucidated; the hydrothermal treatments conducted without stirring in an alkaline media (5 M KOH) using the same reaction precursors resulted in the crystallization of micron-sized (averaging 10 μm) SrZrO_3_ particles with cuboidal morphology [21]. On the contrary, our results depicted that continuous stirring (130 rpm) of the hydrothermal media remarkably improved the size reduction in the cuboidal-like SrZrO_3_ particles, which were produced free of reaction by-products, namely SrCO_3_, at 200 °C for 4 h. The monodispersed SrZrO_3_ particles had a unimodal particle size distribution with an average size of 3.8 μm, as seen in the inlet graph of Figure 7a. Under these conditions, the continuous agitation caused a homogeneous distribution of solute dissolved in the alkaline solvent.

On the contrary, a marked particle reduction occurred on the perovskite particles produced when the Ti^4+^ pasty gel was stoichiometrically introduced into the reaction system. Above Ti^4+^ contents of 5.0 mol%, the single phase SrZr_0.95_Ti_0.05_O_3_ powders exhibited a mean particle size of 141.2 nm. The morphology resembling cuboidal-shaped particles forming some irregularly shaped agglomerates, Figure 7b. Furthermore, the formation of a noticeable number of fine meso-crystals (average size 82.6 nm) with a pseudocuboidal shape were formed together with a large amount of cubic-like shaped large particles having an average size of 175.5 nm, Figure 7c. It deserves to emphasize that these particles were formed using the content of 30 mol% Ti^4+^, and the FE-SEM observation agrees with the PXRD results which revealed the coexistence of two SrZr_1-x_Ti_x_O_3_ solutions with different Ti^4+^ content (Figure 3a). Likewise, the difference on the Zr_(Lα)_ and Ti_(Kα)_ peak intensities between the fine sized pseudocuboidal and large cubic-like particles confirmed the variation on the bulk chemical composition of the SS1 and SS2′ (as seen in Figure S3b in SSIF). Based on the Rietveld refinement analyses, their chemical formulas correspond to SrZr_0.9_Ti_0.1_O_3_ and SrZr_0.70_Ti_0.3_O_3_, respectively.

The pseudocubic meso-crystals were formed preferentially under hydrothermal stirring conditions with Ti^4+^ contents over 50 mol%. Generally, the new pseudocubic-shaped meso-crystals were monodispersed and exhibited a unimodal size distribution with an average size of 172.2 nm. Furthermore, the single-phase SrZr_0.5_Ti_0.5_O_3_ (SS2) cubic structured meso-crystals were likely formed from tiny particles self-assembled via a 3D hierarchical architecture; this assumption was inferred from the roughness surface of the meso-crystals shown in Figure 7d. Additionally, the FE-SEM observations conducted on the hydrothermally produced single phase SrTiO_3_ meso-crystals (see Figure 7e), allowed to infer that these meso-crystals underwent a crystallization process analogous to that observed on samples containing low contents of Ti^4+^ up to 50.0 mol%. Thus, the meso-crystals solely prepared with Ti(OH)_4_^0^ gel had a slight reduction in their particle size, which averaged 147.5 nm. According to the experimental results, it can be inferred that the chemical compositional variation in the crystalline phases produced (SS1, SS2′ and SS2) and the particle coarsening differences are strongly dependent on the Ti(OH)_4_^0^ gel content variation in the hydrothermal treatments conducted under vigorous agitation. Details of the crystallization mechanism and its correlation with the precursor gels (Zr(OH)_4_^0^ and Ti(OH)_4_^0^) are discussed in the following Section 3.4.

### 3.4. Crystallization of SrZr_1-x_Ti_x_O_3_ and SrTi_1-x_Zr_x_O_3_ Meso-Crystals under Alkaline Hydrothermal Conditions

HR-TEM observations and compositional EDS analyses systematically investigated the crystallization process for both SrZr_1-x_Ti_x_O_3_ and SrTi_1-x_Zr_x_O_3_ promoted under hydrothermal treatment conducted with vigorous fluid agitation, according to the differences in morphology revealed by the FE-SEM analyses (Figure 7, and Figure S4 in the SSI), the growth process of the meso-crystals depends on the Ti(OH)_4_^0^ gel. This inference is supported by the remarkable reduction in either Zr^4+^ rich orthorhombic or cubic structured particles preferentially formed on the hydrothermal treatments conducted with compositional variations between 7.5 mol and 50.0 mol% of Ti^4+^. Indeed, some of the present authors reported the differences in the chemical reactivity of metal transition hydroxide gels in the alkaline hydrothermal media [20,21]. It deserves to emphasize that the crystallization of cuboidal-shaped SrZrO_3_ particles rapidly occurred using powders of SrSO_4_ and Zr(OH)_4_^0^ dried gel in a 5 M KOH solution at 240 °C for 24 h, in comparison with an analogous reaction system employing a Zr(OH)_4_^0^ coprecipitated gel. The pasty hydroxide gel undergoes a dehydration process that markedly hinders the SrSO_4_ dissolution; consequently, the bulk solute saturation proceeded slowly, triggering the nucleation of flower-shaped SrZrO_3_ crystals. These crystals grew epitaxially along the [111] direction, resulting in large particles (mean size of 60 μm) for reaction intervals of 96 h [21].

**Figure 8:**
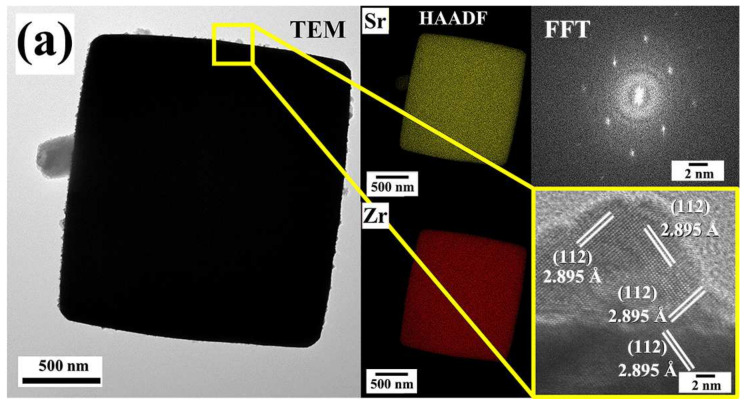

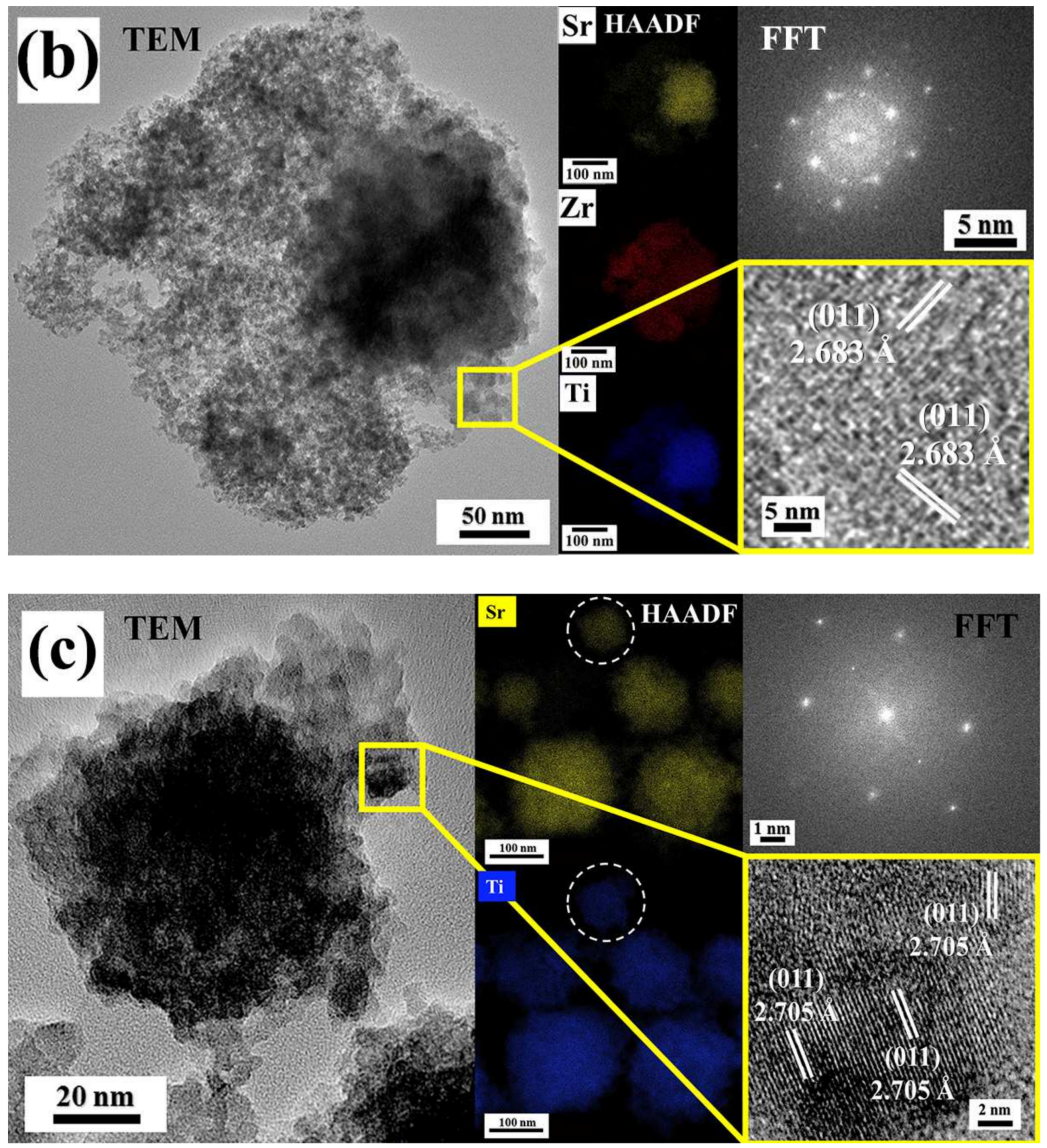
TEM micrographs of the SS SrZr_1-x_Ti_x_O_3_ and SrTi_1-x_Zr_x_O_3_ particles, obtained under hydrothermal conditions at 200 °C in a 5 M KOH for 4 h. The images correspond to single-phase samples of (**a**) SrZrO_3_, (**b**) SrZr_0.5_Ti_0.5_O_3_ and (**c**) SrTiO_3_.

By these results, we infer that the vigorous fluid agitation accelerated the SrSO_4_ dissolution and the pasty Zr(OH)_4_^0^ gel dehydration, consequently triggering a rapid solute supersaturation within 4 h at a relatively low temperature of 200 °C, which rapidly provoked the crystallization of SrZrO_3_ cuboidal-shaped monodispersed crystals. The structural details determined by the TEM diffraction spot image indicated that the hydrothermally prepared SrZrO_3_ crystals are single crystals in nature (Figure 8a). In contrast, the HR-TEM images depicted their high crystallinity, as confirmed by the 2D finger lattice atomic distribution. Interestingly, the observation conducted at the crystal edge in areas exhibiting SrZrO_3_ fine particles grown on the surface indicated that the micron-sized SrZrO_3_ single crystals grew epitaxially along the direction of the plane (112) that, corresponding to the lattice plane distance of 2.895 Å of the orthorhombic structure (card ICDD 70-0283). However, the dissolution-crystallization mechanism provoked the SS1 and SS2 perovskite structured particles, irrespective of the Ti(OH)_4_•4.5H_2_O gel content. Likewise, the reactivity of the Zr(OH)_4_^0^ precursor gel seemingly proceeded faster when compared with the sample prepared solely with Zr(OH)_4_•9.64H_2_O. The bulk alkalinity of the solvent fluid remains high when the Ti^4+^ concentration varies because the alkalinity reduction caused by the large water content released by the Zr(OH)_4_•9.64H_2_O dissolution is progressively hindered. Therefore, a specific reaction steady-state reaction is reached, triggering an accelerated solute supersaturation state that results in a fast crystallization of orthorhombic (SS1) and cubic (SS2′), or solely the SS2 meso-crystals. Consequently, the primary crystal growth is hindered, as revealed by the HR-TEM micrographs of the single-phase SrZr_0.5_Ti_0.5_O_3_ and SrTiO_3_ fine particles shown in Figure 8b and Figure 8c, respectively. These images also revealed features regarding the self-assembly process for primary anhedral nanosized crystals (20–45 nm), which proceeds along the {011} crystallographic planes of the cubic structure. We surmise that their surface facets favor the fine crystals assembly with (011) indexes, seemingly exhibiting low surface energy, allowing them to coalesce rapidly.

Consequently, the 3D hierarchical assembly produces highly crystalline agglomerates with pseudocubic morphology. This inference is supported by 2D finger lattice atomic distribution images and FFT diffraction patterns, as shown in Figure 8b,c. These results revealed that the single-phase cubic structured SrZr_0.5_Ti_0.5_O_3_ and SrTiO_3_ primary crystals grew along (011) planes and subsequently coalesced to form the nanosized (140–175 nm) meso-crystals agglomerates, which exhibited single crystal areas as revealed by the FFT diffraction patterns. Interestingly, the 3D assembly process irrespectively proceeded by varying the Ti^4+^ content in the 7.5–100 mol% range. According to these results, we inferred that under hydrothermal conditions, the continuous vigorous stirring of the alkaline fluid triggered a homogeneous dispersion of the raw precursors (SrSO_4_ powder and the mixture of the pasty gel species), yielding a controlled dissolution-crystallization reaction carried out in a single step to synthesize various perovskite solid solution in the binary system SrZrO_3_–SrTiO_3_. Indeed, the proposed soft chemistry process is faster than that even conducted under highly alkaline 5 M NaOH fluid to produce SrZrO_3_ without stirring under hydrothermal conditions [21].

## 4. Conclusions

A systematic investigation directed toward synthesizing powders with various Zr^4+^ and Ti^4+^ ions contents was satisfactorily conducted via a single-step reaction under hydrothermal conditions. The particles of SrZr_1-x_Ti_x_O_3_ and SrTi_1-x_Zr_x_O_3_ solid solutions rapidly crystallized at 200 °C for 4 h after the dissolution of low-grade SrSO_4_ mineral coupled with the mixture of hydrous Zr^4+^ and Ti^4+^ gels. A dependence on the stability of either crystalline orthorhombic or cubic structured phases occurred on the processed particles against the content of Ti^4+^. The synthesis of the solid solutions was boosted by the vigorous stirring of the alkaline 5 M KOH media, which triggered a massive dissolution–crystallization mechanism of ionic species under alkaline hydrothermal conditions. However, the solvent alkalinity was affected by the Zr(OH)_4_•9.64H_2_O dehydration causing a specific steady-state equilibrium for triggering the solute saturation that achieved simultaneous SS1 and SS2′ fine particle crystallization in the range between 7.5 and 47.5 mol% Ti^4+^.

The rapid dehydration of the Ti-gel in the reaction system reduced the mesocrystals size for the orthorhombic (SS1) and cubic (SS2′) structured solid solutions, resulting in pseudocubic-shaped mesocrystals with sizes varying between 141.0 and 175.5 nm. These mesocrystals were produced via a 3D hierarchical self-assembly process of fine crystals (10.0 nm to 20.0 nm), which occurs along the crystallographic planes {011}, leading to the pseudocubic-shaped agglomerate formation. According to the present results, research must be focused on the electrical and ferroelectric properties of specific chemical compositions for potential industrial application. The soft chemistry hydrothermal processing proposed here might be employed for synthesizing other inorganic perovskite binary systems to tailor solid solutions with enhanced functional properties.

## Data Availability

The data supporting this study’s findings are available from the corresponding author upon reasonable request.

## Supplementary Materials

The following supporting information can be downloaded at: https://www.mdpi.com/article/10.3390/nano13152195/s1, Figure S1: Selected Rietveld refinement plots of powder samples prepared at 200 °C for 4 h with 5 M KOH stirred at 130 rpm, using different Ti4+-gel contents of (a) 0.0, (b) 30.0, (c) 50.0, (d) 80.0 and (e) 100.0 mol% Ti, respectively; Figure S2: Estimated unit cell lattices corresponding to the orthorhombic (a,b) and cubic (c,d) structured solid solutions prepared with different contents of Ti4+, (a) 0.05 mol%, (b,c) 30.0 mol% and (d) 50.0 mol%. The proportional Ti4+ content in each solution is represented in the Zr4+ locations (spatial Wyckoff positions 4c and 4a). The unit cell lattices were plotted using the CIF data file from the Rietveld refinements and the VESTA 3 software (K. Momma and F. Izumi, “VESTA 3 for three-dimensional visualisation of crystal, volumetric and morphology data”, J. Appl. Crystallogr., 44, (2011) 1272–1276. These structures do not represent the real atomic distribution; a superlattice is suggested to be formed to include the stoichiometric amount of the dopant Ti4+ in either orthorhombic or cubic structures.; Figure S3: FE-SEM micrographs of particles of the solid solutions SrZr_1-x_Ti_x_O_3_ and SrTi_1-x_Zr_x_O_3_, obtained under hydrothermal conditions at 200 °C for 4 h, using a reaction medium of 5 M KOH and SrSO4 with different contents of Ti gel (Ti(OH)4•4.5H_2_O), (a) 0.0, (b) 10.0, (c) 15.0, (d) 20.0, (e) 25.0, (f) 30.0, (g) 40.0, (h) 50.0, (i) 75.0 and (j) 100.0 %mol Ti4+; Figure S4: FE-SEM images, mapping of metal elements and EDX spectra of particles corresponding to the solid solutions SrZr_1-x_Ti_x_O_3_ and SrTi_1-x_Zr_x_O_3_, obtained under alkaline hydrothermal conditions at 200 °C for 4 h, using a 5 M KOH solution, SrSO4 and different contents of Ti-gel (Ti(OH)4•4.5H_2_O), (a) 15.0, (b) 30.0 and (c) 50.0 %mol Ti.; Table S1: Strontium titanate cubic structure atomic coordinates associated with the Pm3m, 221, space group. These were used to carry out the Rietveld refinements by TOPAS 4.2 software; spatial locations were reported in the CIF file ICDD card no. 40–1500; Table S2: Strontium zirconate orthorhombic structure atomic coordinates (space group Pbnm, 62) were used for Rietveld refinements by TOPAS 4.2 software; spatial locations were reported in CIF file ICDD card no. 70-0283; Table S3: Summary of experiments conducted to prepare powders of SrZr_1-x_Ti_x_O_3_ and SrTi_1-x_Zr_x_O_3_ under hydrothermal conditions at 200 °C for 4 h.
