# Supplementary material for: Facile Preparation of SrZr1-xTixO3 and SrTi1-xZrxO3 Fine Particles Assisted by Dehydration of Zr4+ and Ti4+ Gels under Hydrothermal Conditions
Source: Nanomaterials (Basel). 2023 Jul 28;13(15):2195. doi: 10.3390/nano13152195 (PMC10420865; doi:10.3390/nano13152195)
Supplement: Supplementary file 1 [file nanomaterials-13-02195-s001.zip › nanomaterials-2518212-supplementary.Update Zully_ s email pdf.pdf]

*Supplementary Supporting Information*

# Facile Preparation of $SrZr_{1-x}Ti_xO_3$ and $SrTi_{1-x}Zr_xO_3$ Fine Particles Assisted by Dehydration of $Zr^{4+}$ and $Ti^{4+}$ Gels under Hydrothermal Conditions

**José Remigio Quiñones-Gurrola [1], Juan Carlos Rendón-Angeles [1,*], Zully Matamoros-Veloza [2], Jorge López-Cuevas [1], Roberto Pérez-Garibay [1] and Kazumichi Yanagisawa [3]**

[1] Centre for Research and Advanced Studies of the National Polytechnic Institute, Saltillo Campus,
Ramos Arizpe 25900, Mexico; jose.quinones@cinvestav.edu.mx (J.R.Q.-G.); jorge.lopez@cinvestav.edu.mx (J.L.-C.); roberto.perez@cinvestav.edu.mx (R.P.-G.)

[2] Tecnológico Nacional de México/(I.T. Saltillo), Technological Institute of Saltillo, Graduate Division, Saltillo 25280, Mexico; zully.mv2@saltillo.tecnm.mx

[3] Research Laboratory of Hydrothermal Chemistry, Faculty of Science, Kochi University, Kochi 780-8073, Japan; yanagi@kochi-u.ac.jp

* Correspondence: jcarlos.rendon@cinvestav.edu.mx; Tel.: +52-(844)-438-9600

**Citation:** Quiñones-Gurrola, J.R.; Rendón-Angeles, J.C.; Matamoros-Veloza, Z.; López-Cuevas, J.; Pérez-Garibay, R.; Yanagisawa, K. Facile Preparation of $SrZr_{1-x}Ti_xO_3$ and $SrTi_{1-x}Zr_xO_3$ Fine Particles Assisted by Dehydration of $Zr^{4+}$ and $Ti^{4+}$ Gels under Hydrothermal Conditions. *Nanomaterials* **2023**, *13*, 2195. https://doi.org/10.3390/nano13152195

Academic Editor: Meiwen Cao

Received: 6 July 2023
Revized: 20 July 2023
Accepted: 21 July 2023
Published: 28 July 2023

**Copyright:** © 2023 by the authors. Licensee MDPI, Basel, Switzerland. This article is an open access article distributed under the terms and conditions of the Creative Commons Attribution (CC BY) license (https://creativecommons.org/licenses/by/4.0/).

This document contains supplementary data on the details of the Rietveld refinement. Likewise, the SEM micrographs corresponding to the particles of the solid solutions $SrZr_{1-x}Ti_xO_3$ and $SrTi_{1-x}Zr_xO_3$ are included to address the analysis of morphology and crystallisation.

***S1. Rietveld Refinement details, refinement plots and selected orthorhombic and cubic unitcell lattices.***

The cards corresponding to the crystalline phases determined in the reaction products from the XRD patterns are shown in Tables S2–S6. These cards are from the COD 2014 database, which runs with the High Score Plus Panalityca software 3.0e. The designed algorithm program for structural refinement includes all crystallographic data listed in Tables S2–S6. The algorithm is based on a 10-coefficient shifted Chebyshev polynomial function that calculates the XRD pattern background, and the pseudo-Voigt function fits the peak shape profile. The algorithm calculates the unit lattice cell parameters, the isotropic thermal displacement, the crystallite size, and the by-product phase content. Typical plots obtained by the refinement algorithm that considers the reaction by-product phases are portrayed in Figure S1.

**Table S1**. Strontium titanate cubic structure atomic coordinates associated with the *Pm3m*, 221, space group. These were used to carry out the Rietveld refinements by TOPAS 4.2 software; spatial locations were reported in the CIF file ICDD card no. 40-1500.

| **Element identification** | **Wyckoff position** | **Occupation** | **Spatial coordinates ($SrTiO_3$)** | | |
|---|---|---|---|---|---|
| | | | **x** | **y** | **z** |
| Sr | 4c | 1 | 0.5 | 0.5 | 0.5 |
| Zr | 4a | 1-X | 0.0 | 0.0 | 0.0 |
| Ti | 4a | X | 0.0 | 0.0 | 0.0 |
| O | 8c | 1 | 0.5 | 0.0 | 0.0 |

**Table S2**. Strontium zirconate orthorhombic structure atomic coordinates (space group *Pbnm*, 62) were used for Rietveld refinements by TOPAS 4.2 software; spatial locations were reported in CIF file ICDD card no. 70-0283.

| **Element identification** | **Wyckoff position** | **Occupation** | **Spatial coordinates ($SrZrO_3$)** | | |
|---|---|---|---|---|---|
| | | | **x** | **y** | **z** |
| Sr | 8d | 1 | 0.9916 | 0.0123 | 0.25 |
| Zr | 4c | 1-X | 0.5 | 0.0 | 0.0 |
| Ti | 4c | X | 0.5 | 0.0 | 0.0 |
| O1 | 4b | 1 | 0.0586 | 0.4687 | 0.25 |
| O2 | 4a | 1 | 0.713 | 0.288 | 0.0371 |

Plots calculated from the structural Rietveld refinement analyses:

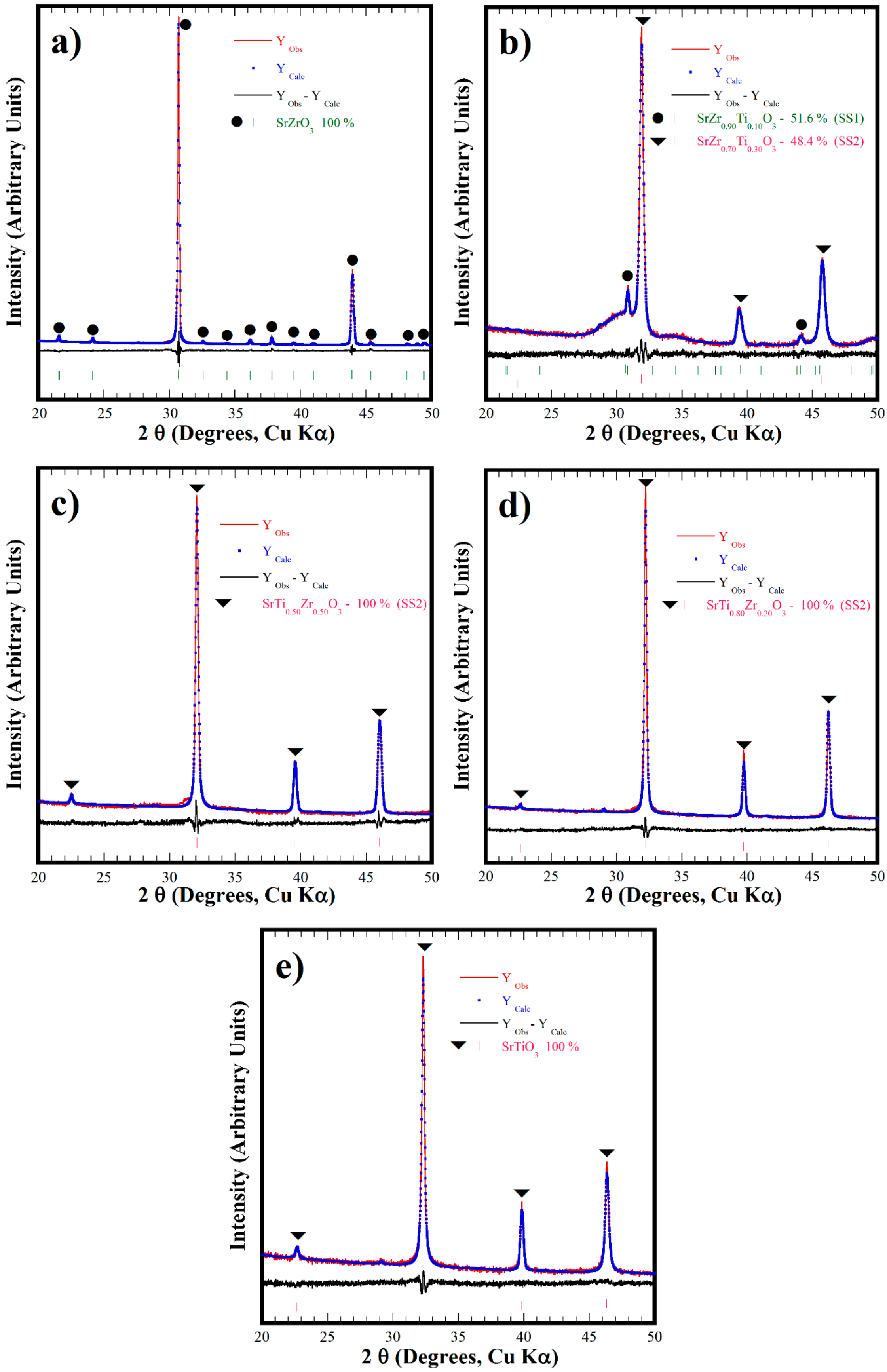


**Figure S1** Selected Rietveld refinement plots of powder samples prepared at 200 ºC for 4 h with 5 M KOH stirred at 130 rpm, using different $Ti^{4+}$-gel contents of a) 0.0, b) 30.0, c) 50.0, d) 80.0 and e) 100.0 mol% Ti, respectively.

***Unit cell lattice example of the perovskite orthorhombic and cubic phases produced under hydrothermal conditions.***

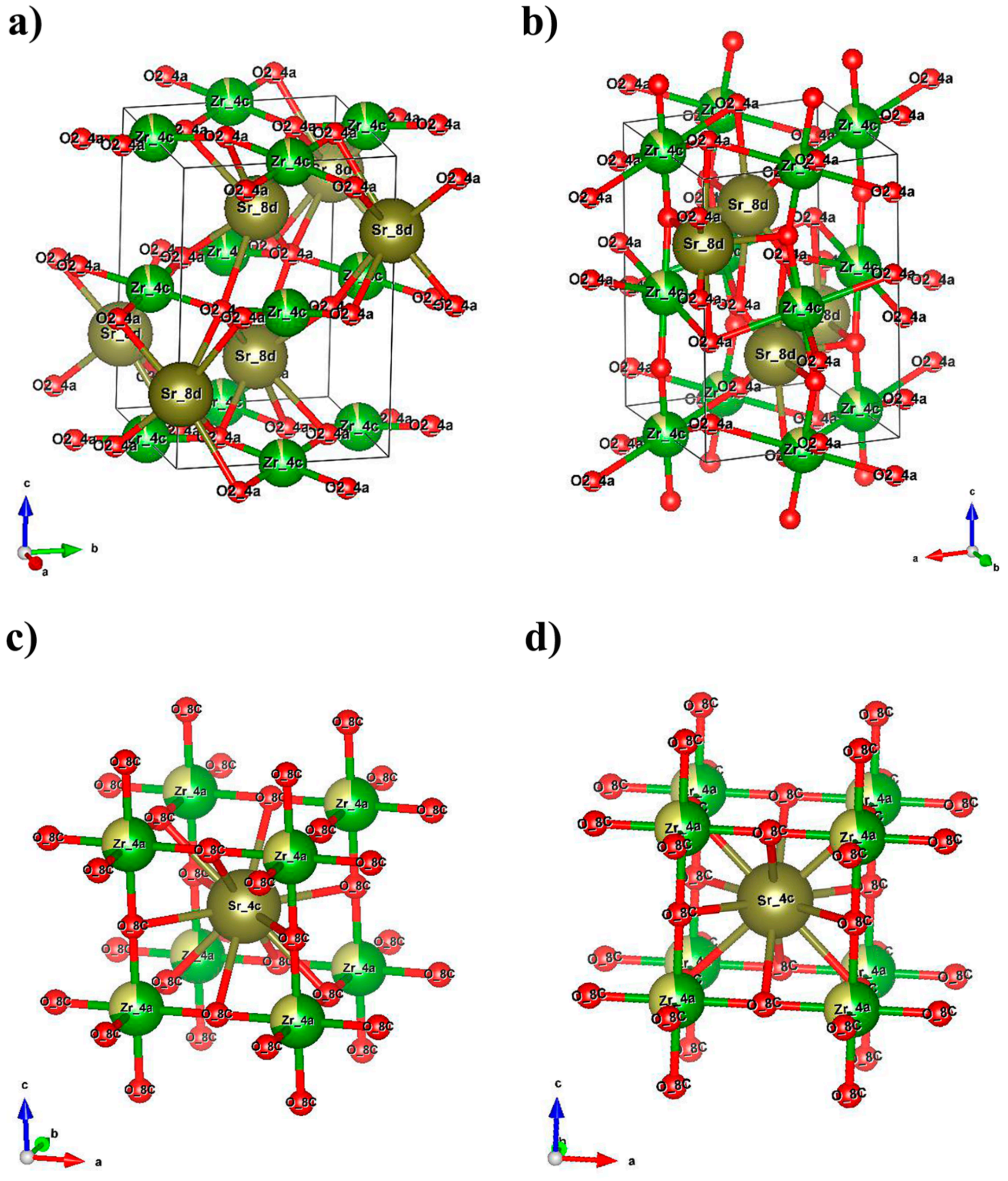


**Figure S2** Estimated unit cell lattices corresponding to the orthorhombic (a,b) and cubic (c,d) structured solid solutions prepared with different contents of $Ti^{4+}$, (a) 0.05 mol%, (b, c) 30.0 mol% and (d) 50.0 mol%. The proportional $Ti^{4+}$ content in each solution is represented in the $Zr^{4+}$ locations (spatial Wyckoff positions 4c and 4a). The unit cell lattices were plotted using the CIF data file from the Rietveld refinements and the VESTA 3 software (K. Momma and F. Izumi, "VESTA 3 for three-dimensional visualisation of crystal, volumetric and morphology data," J. Appl. Crystallogr., 44, (2011) 1272-1276. These structures do not represent the real atomic distribution; a superlattice is suggested to be formed to include the stoichiometric amount of the dopant $Ti^{4+}$ in either orthorhombic or cubic structures.

***S2. Chemical equilibrium promoted in alkaline hydrothermal conditions for synthesising solid solutions particles in the binary system $SrZrO_3$–$SrTiO_3$.***

Chemical equilibrium proposed from the PXRD analyses conducted on powdered specimens produced varying the $Ti^{4+}$-gel precursor content; these equilibria trigger the formation of nanosized particles under alkaline hydrothermal conditions with continuous fluid stirring at 130 rpm. The sub-index abbreviations in the chemical equilibria, "*ort*" and "*cub*", correspond to orthorhombic and cubic crystalline structures, and "*mc*" to meso-crystals.

$$SrSO_{4(s)} + (1\text{-}x)\ Zr(OH)_4^{-}{}_{(aq} + x\ Ti(OH)_4^{-}{}_{(aq)} + 5KOH_{(aq)}$$

$$\rightarrow SrZr_{1\text{-}x}Ti_xO_{3(mc,ort)} + K_2SO_{4(aq)} + 3KOH_{(l)} + 3H_2O \quad \text{(S1)}$$

$$SrSO_{4(s)} + (1\text{-}[x+y])\ Zr(OH)_4^{-}{}_{(aq)} + (1\text{-}[x+y])\ Ti(OH)_4^{-}{}_{(aq)} + 5KOH_{(aq)}$$

$$\rightarrow [1\text{-}a]\ SrZr_{1\text{-}x}Ti_xO_{3(mc,ort)} + [a]\ SrZr_{1\text{-}y}Ti_yO_{3(mc,cub)} + K_2SO_{4(aq)} + 3KOH_{(l)} + 3H_2O \quad \text{(S2)}$$

$$SrSO_{4(pow)} + x\ Zr(OH)_4^{-}{}_{(aq)} + (1\text{-}x)\ Ti(OH)_4^{-}{}_{(aq)} + 5KOH_{(aq)}$$

$$\rightarrow SrTi_{1\text{-}x}Zr_xO_{3(mc,cub)} + K_2SO_{4(aq)} + 3KOH_{(l)} + 3H_2O \quad \text{(S3)}$$

**Table S3** Summary of experiments conducted to prepare powders of $SrZr_{1-x}Ti_xO_3$ and $SrTi_{1-x}Zr_xO_3$ under hydrothermal conditions at 200 ºC for 4 h.

| Sample ID | Ti / Zr % at | Crystalline Phases produced | Crystalline structure | Crystalline phases % | Lattice parameters | | | Crystallite size (nm) | Cell Volume (Å$^3$) | Bond length (Å) | | | $R_{wp}$ (%) | GoF ($\chi^2$) |
|---|---|---|---|---|---|---|---|---|---|---|---|---|---|---|
| | | | | | a (Å)[a] | b (Å)[a] | c (Å)[a] | | | Sr-O | Ti-O | Zr-O | | |
| SZG22R | 100 | $SrZrO_3$ | *Pbnm* | 100 | 5.8200 (1) | 5.8207 (1) | 8.2293 (2) | 100.83 (5) | 278.78 (12) | 2.161 (3) | - | 2.024 (3) | 6.06 | 5.164 |
| SZG46R | 5 /100 | $SrZr_{0.95}Ti_{0.05}O_3$ | *Pbnm* | 100 | 5.8191 (1) | 5.8120 (2) | 8.2314 (3) | 115.37 (7) | 278.39 (17) | 1.819 (1) | 2.209 (1) | 2.209 (1) | 4.71 | 4.19 |
| SZG47R | 7.5 / 92.5 | $SrZr_{0.925}Ti_{0.075}O_3$ | *Pm3m* | 8.56 | 3.9889 (5) | - | - | 66.67 (21) | 63.59 (2) | 2.820 (1) | 1.994 (1) | 1.994 (1) | 3.04 | 2.95 |
| | | $SrZr_{0.93}Ti_{0.07}O_3$ | *Pbnm* | 91.44 | 5.8262 (7) | 5.8147 (2) | 8.2184 (4) | 254.46 (15) | 278.42 (3) | 2.541 (1) | 2.061 (1) | 2.061 (1) | | |
| SZG37R | 10 / 90 | $SrZr_{0.90}Ti_{0.10}O_3$ | *Pm3m* | 14.86 | 3.9916 (9) | - | - | 28.89 (11) | 63.47(9) | 2.822 (2) | 1.995 (2) | 1.995 (2) | 3.38 | 3.19 |
| | | $SrZr_{0.928}Ti_{0.0702}O_3$ | *Pbnm* | 85.14 | 5.8059 (13) | 5.8036 (14) | 8.2192 (14) | 103.49 (24) | 278.38 (12) | 2.353 (2) | 2.035 (2) | 2.035 (2) | | |
| SZG48R | 12.5 / 87.5 | $SrZr_{0.875}Ti_{0.125}O_3$ | *Pm3m* | 15.76 | 3.9773 (3) | - | - | 60.64 (22) | 62.92 (1) | 2.812 (1) | 1.988 (1) | 1.988 (1) | 2.90 | 3.02 |
| | | $SrZr_{0.925}Ti_{0.075}O_3$ | *Pbnm* | 84.21 | 5.8025 (8) | 5.8272 (8) | 8.2195 (6) | 208.56 (21) | 277.92 (6) | 2.207 (1) | 1.999 (1) | 1.999 (1) | | |
| SZG49R | 15 / 85 | $SrZr_{0.85}Ti_{0.15}O_3$ | *Pm3m* | 19.4 | 3.9683 (4) | - | - | 17.01 (2) | 62.49 (2) | 2.806 (3) | 1.984 (3) | 1.984 (3) | 2.84 | 3.01 |
| | | $SrZr_{0.92}Ti_{0.08}O_3$ | *Pbnm* | 80.6 | 5.8135 (5) | 5.8092 (5) | 8.2152 (5) | 38.13 (5) | 277.44 (4) | 1.942 (3) | 2.061 (3) | 2.061 (3) | | |
| SZG50R | 17.5 / 82.5 | $SrZr_{0.825}Ti_{0.175}O_3$ | *Pm3m* | 20.14 | 3.9653 (4) | - | - | 122.95 (26) | 62.34 (2) | 2.803 (1) | 1.982 (1) | 1.982 (1) | 2.92 | 3.12 |
| | | $SrZr_{0.918}Ti_{0.082}O_3$ | *Pbnm* | 79.86 | 5.7903 (19) | 5.8154 (14) | 8.2161 (14) | 244.77 (25) | 277.00 (12) | 2.109 (1) | 2.069 (1) | 2.069 (1) | | |
| SZG51R | 20 / 80 | $SrZr_{0.80}Ti_{0.20}O_3$ | *Pm3m* | 21.27 | 3.9653 (2) | - | - | 47.69 (12) | 62.35 (1) | 2.803 (2) | 1.982 (2) | 1.982 (2) | 2.71 | 2.93 |
| | | $SrZr_{0.915}Ti_{0.085}O_3$ | *Pbnm* | 78.73 | 5.8034 (5) | 5.8151 (5) | 8.2151 (5) | 85.89 (19) | 277.24 (4) | 2.315 (2) | 2.105 (2) | 2.105 (2) | | |
| SZG52R | 22.5 / 77.5 | $SrZr_{0.775}Ti_{0.225}O_3$ | *Pm3m* | 29.7 | 3.9612 (3) | - | - | 139.98 (14) | 62.15 (1) | 2.801 (3) | 1.980 (3) | 1.980 (3) | 2.95 | 2.98 |
| | | $SrZr_{0.911}Ti_{0.089}O_3$ | *Pbnm* | 70.3 | 5.7905 (16) | 5.8374 (8) | 8.2068 (8) | 247.27 (14) | 277.40 (12) | 2.474 (3) | 2.075 (3) | 2.075 (3) | | |
| SZG54R | 25 / 75 | $SrZr_{0.75}Ti_{0.25}O_3$ | *Pm3m* | 34.28 | 3.9553 (9) | - | - | 200.86 (24) | 61.87 (4) | 2.796 (4) | 1.977 (4) | 1.977 (4) | 3.01 | 3.05 |
| | | $SrZr_{0.91}Ti_{0.09}O_3$ | *Pbnm* | 65.72 | 5.7988 (15) | 5.8174 (17) | 8.2097 (17) | 231.73 (19) | 276.94 (12) | 1.896 (4) | 2.094 (4) | 2.094 (4) | | |
| SZG55R | 27.5 / 72.5 | $SrZr_{0.725}Ti_{0275}O_3$ | *Pm3m* | 41.45 | 3.9510 (5) | - | - | 135.16 (50) | 61.67 (2) | 2.793 (1) | 1.975 (8) | 1.975 (8) | 3.07 | 3.13 |
| | | $SrZr_{0.905}Ti_{0.095}O_3$ | *Pbnm* | 58.55 | 5.8042 (13) | 5.7925 (13) | 8.2082 (12) | 252.25 (22) | 276.21 (1) | 1.646 (1) | 2.148 (8) | 2.148 (8) | | |
| SZG39R | 30 / 70 | $SrZr_{0.70}Ti_{0.30}O_3$ | *Pm3m* | 48.4 | 3.9649 (1) | - | - | 56.97 (8) | 61.63 (7) | 2.803 (6) | 1.982 (6) | 1.982 (6) | 3.61 | 3.20 |
| | | $SrZr_{0.9}Ti_{0.1}O_3$ | *Pbnm* | 51.6 | 5.7150 (46) | 5.8608 (18) | 8.2125 (18) | 78.04 (14) | 276.07 (3) | 2.053 (6) | 2.060 (6) | 2.060 (6) | | |
| SZG57R | 32.5 / 67.5 | $SrZr_{0.675}Ti_{0.325}O_3$ | *Pm3m* | 50.13 | 3.9492 (6) | - | - | 103.79 (24) | 61.59 (9) | 2.792 (2) | 1.974 (2) | 1.974 (2) | 3.19 | 3.24 |
| | | $SrZr_{0.9}Ti_{0.1}O_3$ | *Pbnm* | 49.87 | 5.7687 (12) | 5.8315 (15) | 8.1967 (15) | 246.31 (22) | 275.74 (1) | 1.712 (2) | 2.123 (2) | 2.123 (2) | | |

**Table S3 continue.**

| Sample ID | Ti / Zr % at | Crystalline phases produced | Crystalline structure | Crystalline phases % | Lattice parameters | | | Crystallite size (nm) Rietveld | Cell volume (Å$^3$) | Bond length (Å) | | | Rwp (%) | GoF ($\chi^2$) |
|---|---|---|---|---|---|---|---|---|---|---|---|---|---|---|
| | | | | | a (Å)[a] | b (Å)[a] | c (Å)[a] | | | Sr-O | Ti-O | Zr-O | | |
| SZG58R | 35 / 65 | $SrZr_{0.65}Ti_{0.35}O_3$ | *Pm3m* | 51.68 | 3.9466 (3) | - | - | 93.81 (26) | 61.47 (1) | 2.790 (4) | 1.973 (2) | 1.973 (2) | 3.12 | 3.16 |
| | | $SrZr_{0.9}Ti_{0.1}O_3$ | *Pbnm* | 48.32 | 5.7537 (7) | 5.8267 (7) | 8.1942 (7) | 106.60 (85) | 274.89 (1) | 1.862 (4) | 2.164 (2) | 2.164 (2) | | |
| SZG59R | 37.5 / 62.5 | $SrZr_{0.625}Ti_{0.375}O_3$ | *Pm3m* | 53.42 | 3.9433 (2) | - | - | 92.41 (17) | 61.32 (1) | 2.788 (4) | 1.971 (2) | 1.971 (2) | 2.98 | 3.03 |
| | | $SrZr_{0.9}Ti_{0.1}O_3$ | *Pbnm* | 46.58 | 5.7451 (5) | 5.8138 (5) | 8.1991 (5) | 228.98 (27) | 273.86 (1) | 1.853 (4) | 2.167 (2) | 2.167 (2) | | |
| SZG60R | 40 / 60 | $SrZr_{0.60}Ti_{0.40}O_3$ | *Pm3m* | 55.57 | 3.9384 (2) | - | - | 54.54 (6) | 61.09 (1) | 2.784 (4) | 1.969 (3) | 1.969 (3) | 3.23 | 3.29 |
| | | $SrZr_{0.9}Ti_{0.1}O_3$ | *Pbnm* | 44.43 | 5.7445 (16) | 5.8264 (7) | 8.1958 (7) | 40.29 (12) | 273.39 (2) | 1.221 (4) | 1.779 (3) | 1.779 (3) | | |
| SZG61R | 42.5 / 57.5 | $SrZr_{0.575}Ti_{0.425}O_3$ | *Pm3m* | 60.05 | 3.9417 (3) | - | - | 42.57 (3) | 61.24 (1) | 2.787 (7) | 1.970 (7) | 1.970 (7) | 3.26 | 3.59 |
| | | $SrZr_{0.895}Ti_{0.105}O_3$ | *Pbnm* | 39.95 | 5.7436 (18) | 5.9838 (6) | 8.1893 (6) | 10.30 (1) | 272.67 (2) | 2.320 (7) | 1.876 (7) | 1.876 (7) | | |
| SZG62R | 45 / 55 | $SrZr_{0.55}Ti_{0.45}O_3$ | *Pm3m* | 74.26 | 3.9356 (1) | - | - | 46.85 (3) | 60.95 (1) | 2.782 (4) | 1.967 (6) | 1.967 (6) | 5.75 | 4.45 |
| | | $SrZr_{0.89}Ti_{0.11}O_3$ | *Pbnm* | 25.74 | 5.7435 (12) | 5.6016 (9) | 8.1882 (9) | 63.32 (2) | 272.26 (3) | 0.714 (4) | 0.668 (6) | 0.668 (6) | | |
| SZG63R | 47.5 / 52.5 | $SrZr_{0.525}Ti_{0.475}O_3$ | *Pm3m* | 85.17 | 3.9354 (1) | - | - | 37.40 (4) | 60.95 (1) | 2.782 (3) | 1.967 (2) | 1.967 (2) | 4.04 | 3.80 |
| | | $SrZr_{0.885}Ti_{0.115}O_3$ | *Pbnm* | 14.83 | 5.7433 (17) | 5.8841 (3) | 8.1066 (3) | 71.61 (65) | 271.91 (3) | 0.410 (3) | 0.720 (2) | 0.720 (2) | | |
| SZG36R | 50 / 50 | $SrTi_{0.5}Zr_{0.5}O_3$ | *Pm3m* | 100 | 3.9439 (10) | - | - | 53.65 (2) | 60.86 (1) | 2.788 (1) | 1.971 (1) | 1.971 (1) | 5.46 | 4.55 |
| SZG35R | 60 / 40 | $SrTi_{0.6}Zr_{0.4}O_3$ | *Pm3m* | 100 | 3.9368 (14) | - | - | 141.70 (9) | 60.81 (1) | 2.783 (1) | 1.968 (1) | 1.968 (1) | 4.93 | 3.97 |
| SZG34R | 70 / 30 | $SrTi_{0.7}Zr_{0.3}O_3$ | *Pm3m* | 100 | 3.9296 (11) | - | - | 71.72 (2) | 60.68 (1) | 2.778 (1) | 1.964 (1) | 1.964 (1) | 4.73 | 3.67 |
| SZG33R | 80 / 20 | $SrTi_{0.8}Zr_{0.2}O_3$ | *Pm3m* | 100 | 3.9254 (6) | - | - | 77.18 (1) | 60.48 (1) | 2.775 (1) | 1.962 (1) | 1.962 (1) | 4.68 | 3.51 |
| SZG32R | 90 / 10 | $SrTi_{0.9}Zr_{0.1}O_3$ | *Pm3m* | 100 | 3.9218 (4) | - | - | 74.28 (1) | 60.32 (1) | 2.773 (1) | 1.960 (1) | 1.960 (1) | 4.90 | 3.59 |
| SZG66R | 100 | $SrTiO_3$ | *Pm3m* | 100 | 3.9172 (12) | - | - | 85.22 (5) | 60.09 (1) | 2.769 (1) | 1.958 (1) | - | 4.04 | 3.25 |
| Strontium zirconate ($SrZrO_3$)[b] | | | *Pbnm* | | 5.786 | 5.815 | 8.196 | | 275.75 | | | | | |
| Strontium titanate ($SrTiO_3$)[c] | | | *Pm3m* | | 3.905 | - | - | | 59.54 | | | | | |

Note: *Pm3m* = Crystalline structure orthorhombic.
*Pbnm* = Crystalline structure cubic.

[a] The lattice parameters “a” were only determined for solid solution $SrTi_{1-x}Zr_xO_3$ and “a”, “b” and “c” were only determined for solid solution $SrZr_{1-x}Ti_xO_{3;\ these}$ values were calculated considering anisotropic lattice strain, particle size and preferential orientation as parameters for the Rietveld refinement analyses.

[b] Strontium zirconate structure ICDD card No. 70-0283.

[c] Strontium titanate structure ICDD card No. 40-1500.

## *S3. Microstructural features of the SS particles prepared under alkaline hydrothermal conditions with vigorous fluid stirring*

Figure S3 shows the morphology of particles obtained at 200 ºC for 4 h in a 5 M KOH solution with different Zr and Ti gels contents. Generally, the micrographs showed remarkable differences in the particle size of the fine mesocrystals produced over $Ti^{4+}$ contents of 15 mol%. In all cases, the microstructural results indicated that secondary phases were not simultaneously produced during the crystallisation of the perovskite-structured orthorhombic (SS1) and cubic (SS2' and SS2) mesocrystals. Furthermore, the mesocrystals morphology is irrespective of the crystalline phase formed (Figures S3a–S3j); the particles exhibited a cubic habit which agrees with the morphological crystalline features of the mineral perovskite specie ($CaTiO_3$). The agglomerated mesocrystals formed by tiny crystals corresponding to the new cubic-structured SS2' phase rich in $Zr^{4+}$ were visible on the FE SEM micrographs of powder samples prepared with $Ti^{4+}$ contents over 20.0 mol% (Figures S3d – S3h). These results are analogous to the SS1 and SS2' amounts calculated by the refinement analyses in Table S3. Therefore, the SS2' mesocrystals were difficult to determine on the samples prepared with low $Ti^{4+}$ contents between 7.5 and 20.0 mol %. In addition, it deserves to emphasise that the SS2' cubic-like shaped particles exhibit a particular surface rugosity, which is visible on the cuboidal-shaped mesocrystals {100} facets. This texture resulted from the tiny crystals' 3D hierarchical self-assembly process, forming the agglomerated SS2' cubic perovskite mesocrystals. On the contrary, the orthorhombic structured SS1 mesocrystals exhibited flat and smooth crystal {100} facets.

Figure S4 shows the results of the chemical compositional analyses determined by the EDX microprobe. The area mode acquisition measurements were conducted on the selected particles, highlighted by the yellow square in each case; for samples prepared with different $Ti^{4+}$ molar concentrations. Furthermore, the area mapping images and energy spectra of both SS1 and SS2' particles are also portrayed in Figs 4a-4c. The differences revealed from these analyses agree with the stoichiometry contents of these crystalline phases used for the Rietveld refinement analyses in each case. In addition, the chemical compositional differences between the orthorhombic (SS1) and cubic (SS2') structured particles were confirmed by the area mapping images and the EDX spectra given in Figs. 4a-4c. Hence, these results confirmed that under hydrothermal conditions with stirring triggered, the simultaneous crystallisation of the mixture of the phases abovementioned.

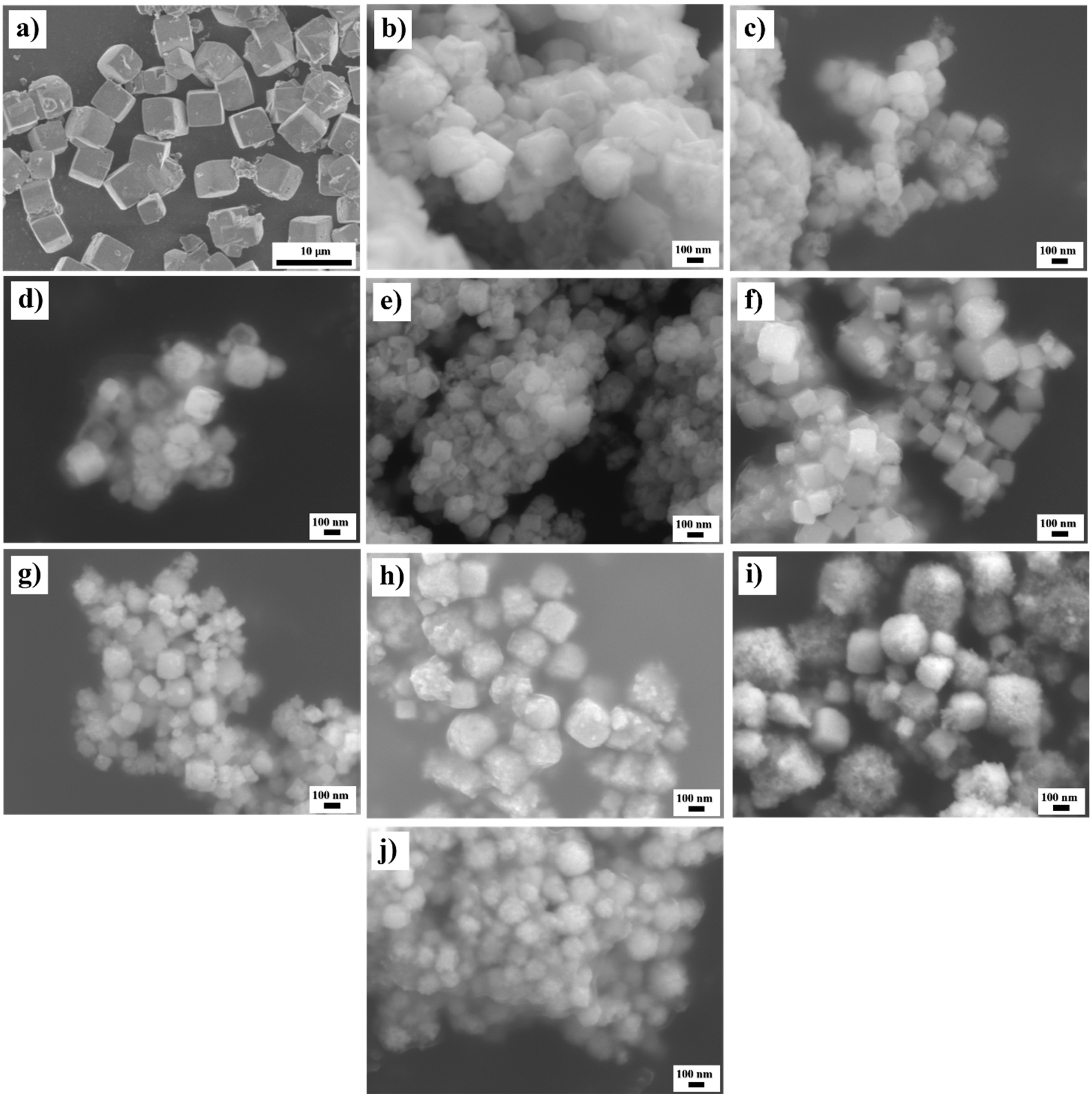


**Figure S3** FE-SEM micrographs of particles of the solid solutions $SrZr_{1-x}Ti_xO_3$ and $SrTi_{1-x}Zr_xO_3$, obtained under hydrothermal conditions at 200 °C for 4 h, using a reaction medium of 5 M KOH and $SrSO_4$ with different contents of Ti gel ($Ti(OH)_4$•$4.5H_2O$), a) 0.0, (b) 10.0, (c) 15.0, (d) 20.0, (e) 25.0, (f) 30.0, (g) 40.0, (h) 50.0, (i) 75.0 and (j) 100.0 %mol $Ti^{4+}$.

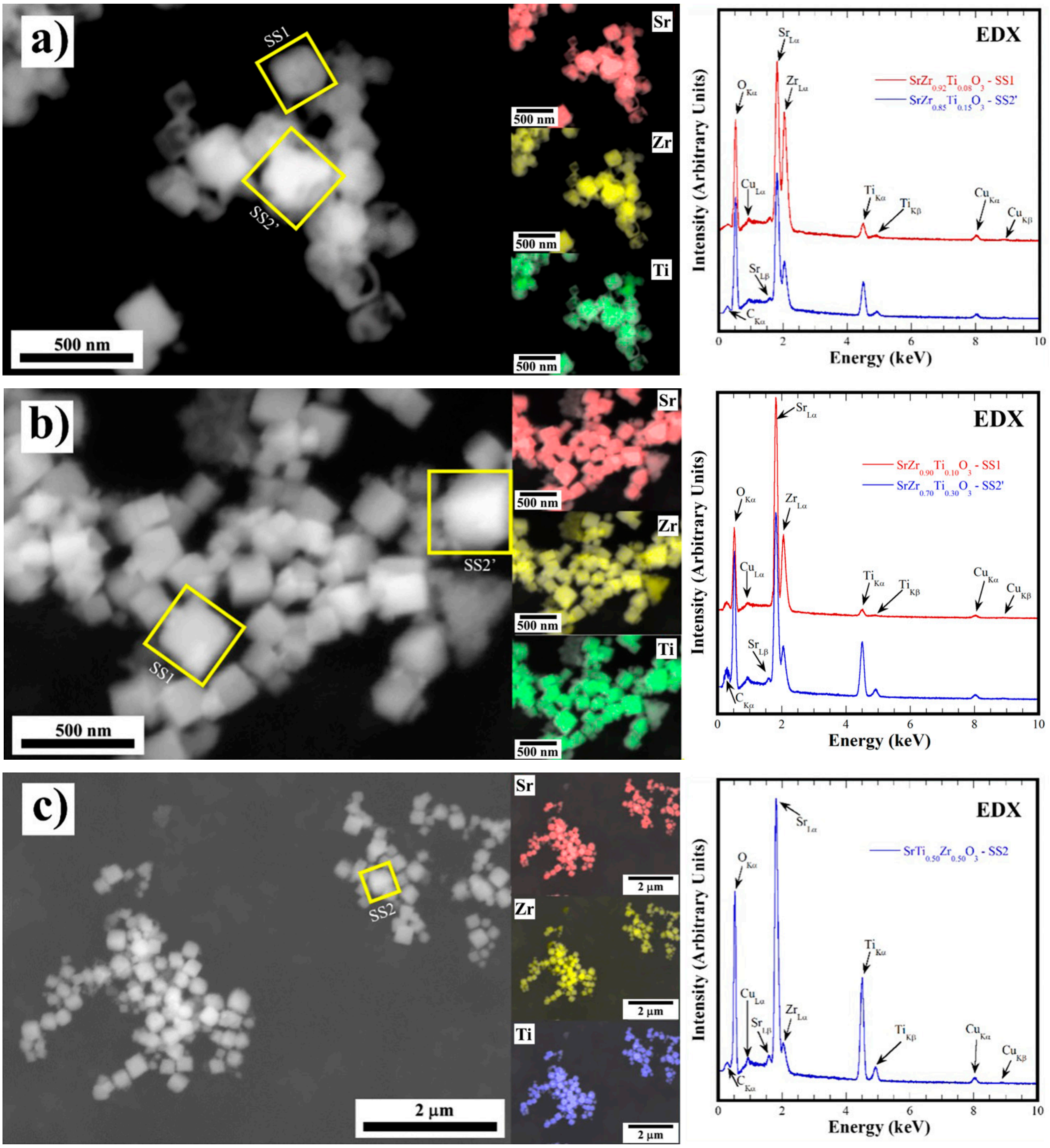


**Figure S4** FE-SEM images, mapping of metal elements and EDX spectra of particles corresponding to the solid solutions $SrZr_{1-x}Ti_xO_3$ and $SrTi_{1-x}Zr_xO_3$, obtained under alkaline hydrothermal conditions at 200 °C for 4 h, using a 5 M KOH solution, $SrSO_4$ and different contents of Ti-gel ($Ti(OH)_4$•$4.5H_2O$), a) 15.0, b) 30.0 and c) 50.0 %mol Ti.
